# Plant-oil-based adhesive formulations induce plant stress responses and enhance resistance of *Chrysanthemum* to western flower thrips

**DOI:** 10.1039/d6ra07230e

**Published:** 2026-10-27

**Authors:** Thijs V. Bierman, Hocelayne P. Fernandes, Sumin Seo, T. Martijn Bezemer, Klaas Vrieling, Ralph Van Zwieten, Thomas E. Kodger, Peter G. L. Klinkhamer, Young H. Choi

**Affiliations:** a Above-Belowground Interactions, Institute of Biology Leiden Leiden The Netherlands t.v.bierman@biology.leidnuniv.nl t.m.bezemer@biology.leidenuniv.nl k.vrieling@biology.leidenuniv.nl p.g.l.klinkhamer@biology.leidenuniv.nl; b Natural Products Laboratory, Institute of Biology Leiden Leiden The Netherlands y.choi@chem.leidenuniv.nl hocelayne.fernandes@qti-services.com s.seo@biology.leidenuniv.nl; c Physical Chemistry and Soft Matter, Agrotechnology & Food Sciences Group, Wageningen University & Research Wageningen The Netherlands ralph.vanzwieten@wur.nl thomas.kodger@wur.nl

## Abstract

We tested whether a new pest control method: sprayable adhesive droplets made from oxidized plant oils (MIX), can physically trap thrips on *Chrysanthemum* leaf surfaces and investigated the effects of spraying plants with MIX on the metabolome of local and systemic leaves in interaction with herbivory of thrips. MIX adhesive did not effectively trap thrips. However, total thrips damage and reproduction were reduced by MIX spraying, especially on leaves in the middle of the shoot (the youngest tissue when sprayed). Regarding metabolomic effects, no interaction between MIX application and thrips herbivory was found. Locally, the levels of several plant-stress related compounds differed between the leaves of MIX-sprayed- and control plants. No systemic effect of spraying was observed in newly grown leaves. Thrips infestation and plant tissue age were themselves also associated with differences in the contents of several metabolites that are linked to herbivore resistance.

## Abstract 2

We investigated whether sprayable adhesive droplets made from oxidized plant oils (MIX) physically trap thrips on *Chrysanthemum* leaves and how MIX spraying and thrips herbivory affect the metabolomes of local and systemic leaves.

## Graphical abstract

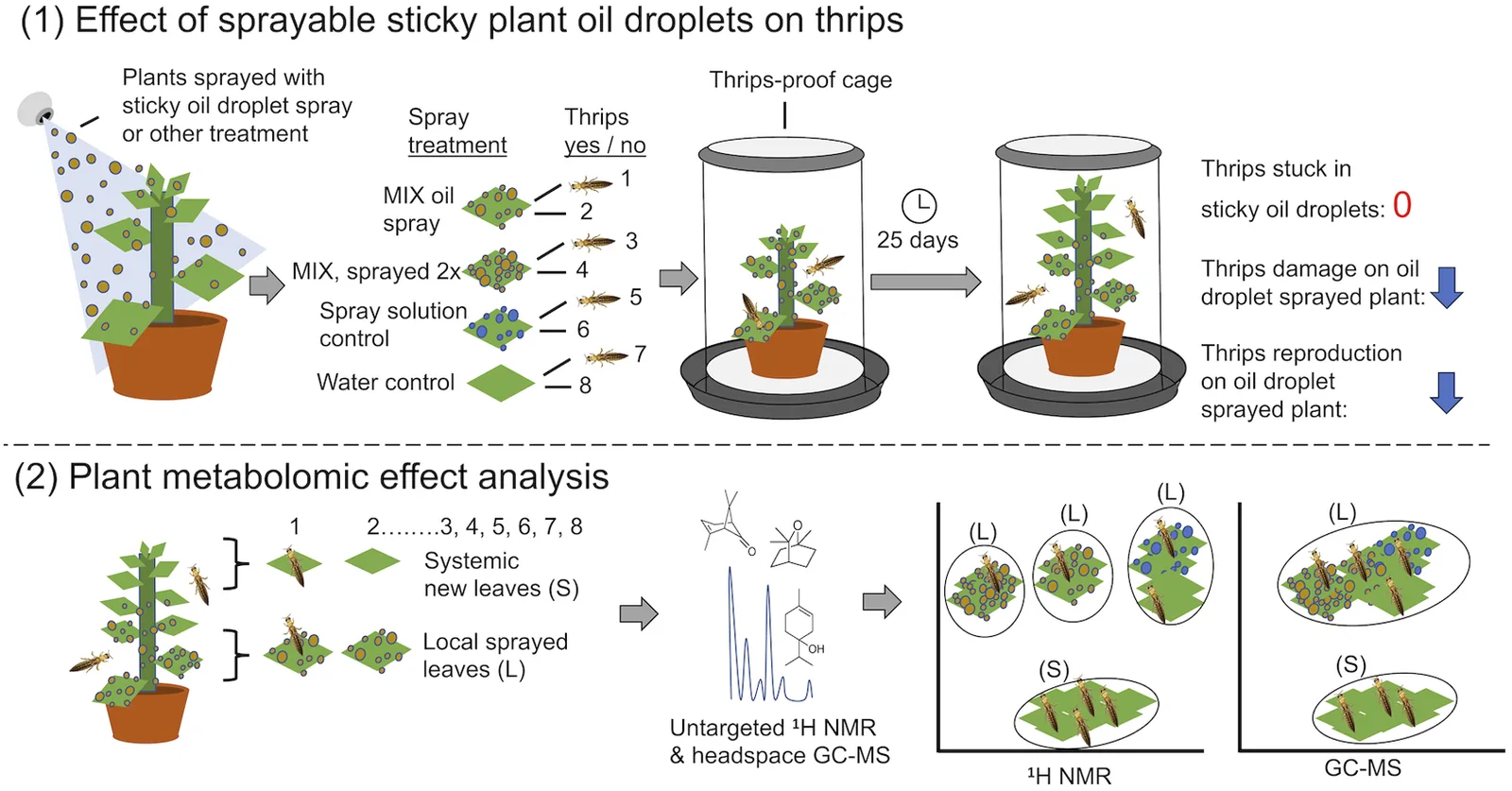

## Introduction

Chrysanthemum (*Chrysanthemum* × *morifolium* [Ramat.] Hemsl.) is a flowering crop of global economic importance with thousands of cultivars in trade.^1^ One of the most important pests in *Chrysanthemum* cultivation is western flower thrips (WFT), *Frankliniella occidentalis* (Pergande), which is responsible for annual agricultural losses worth millions of euros.^2,3^ WFT causes visual damage and reduced or malformed growth by feeding and can act as a vector for several tospoviruses, including *Chrysanthemum* stem necrosis virus and tomato spotted wilt virus.^4,5^ A fast lifecycle, high fecundity, small size, and preference for hiding between plant parts all contribute to why WFT populations are hard to manage.^6^

Control of WFT in *Chrysanthemum* and other crops relies almost exclusively on synthetic pesticides. However, overuse of these chemicals has led to thrips populations becoming resistant^7^ and other negative consequences for human health, the environment, and biodiversity.^8–10^ With pesticide regulations becoming increasingly strict,^8^ more sustainable management methods for thrips are urgently needed. Pest control products made from natural materials with insecticidal or arthropod-repellent properties seem a promising alternative.^11^ For example, *Chrysanthemum cinerariifolium* is used to produce pyrethrum,^12^ a natural insecticide that is effective against a range of arthropod pests,^13^ and plant-based oils have been found effective in repelling thrips and lowering thrips populations.^14–17^

Next to providing direct anti-arthropod effects, the application of natural materials such as plant-based oils can alter plant physiology like growth and appearance and may also influence the plant's resilience to pests and diseases, often *via* chemical changes within plant tissues and emitted volatiles.^18–21^ These responses can be general or very specific, dose responsive and can occur locally or systemically throughout the whole plant. Crop protection methods based on natural materials that impact pests both directly by acting as repellents or feeding deterrents and indirectly *via* systemic induction of plant defenses may be very beneficial to increase crop resilience against thrips and other arthropod pests. Importantly however, pests such as thrips also induce the plant's metabolomic responses.^22–24^ Hence, the overall plant metabolome of *Chrysanthemum* may be affected in two ways: Firstly, since the quantity of herbivore feeding is often linked to the strength of plant defense induction,^25^ protecting plants using natural materials may have consequences for the strength of the systemic response of the plant to thrips infestation. Secondly, the metabolome of plants may respond differently to natural product application depending on whether pests are present or not. Studies that have investigated the three-way interaction between crop protection products, plants, and pests simultaneously on plant chemical defenses are however scarce. Considering the increasing use of natural materials in agriculture and lack of detailed understanding of product–plant–pest interactions, there is a need for knowledge of how crop protection products made from natural materials and herbivory interact on local and systemic levels to shape plant physiology and resilience.^26–28^

In this study, we investigated the thrips control potency and metabolomic effects of a new type of pest control method: a sprayable solution containing naturally sourced chemicals and adhesive droplets made from a mixture of sunflower oil, linseed oil and olive oil (hereafter referred to as MIX).^29^ Compared to the more regular use of plant-based oils as a repellent coating, our method aims to utilize the innate sticky properties of plant-based oils as both a sticky trap and repellent barrier on the plant surface, similar to the adhesive substances and volatiles that are secreted by the glandular trichomes of many vascular plants.^30^ Another aim is to boost plant resilience *via* the induction of plant innate chemical defenses. In previous work, we found that droplets of MIX adhesive and those made of rice germ oil (RGO) can catch thrips and may reduce thrips damage and reproduction when tested on abscised *Chrysanthemum* leaves.^29,31^ More recently, we showed that the RGO formulations, despite not catching many thrips, substantially reduces thrips damage on young *Chrysanthemum* plants and that the effect of thrips presence on the metabolome of sprayed *Chrysanthemum* leaves was less distinguished than on the leaves of water sprayed plants.^32^ Following these observations, we now investigate whether the MIX formulation is more effective to trap thrips on plant surfaces and to reduce thrips damage and population size. In addition, we aimed to further uncover the effects of the application of natural materials on the plant metabolome and their interaction with thrips infestation.

Since several different volatile and non-volatile compounds are likely involved in herbivore resistance in *Chrysanthemum*,^33–35^ we wanted to monitor the effects of MIX application and thrips presence over a broad range of metabolites. Therefore, we used two analytical platforms: proton nuclear magnetic resonance spectroscopy (^1^H NMR) and headspace gas chromatography-mass spectrometry (HS-GC-MS). The integration of these two approaches was expected to provide a comprehensive overview of the metabolic changes resulting from MIX application and thrips infestation. The results we present here provide new insights on which compounds likely play a role in thrips resistance and how natural product application and pest presence interact to influence the levels of these compounds within plants. This knowledge may guide resistance breeding and the effective application of natural materials in pest control.

## Experimental

### Sprayable solutions containing adhesive droplets

Solutions containing adhesive droplets were made from a mixture of 325 ml sunflower oil, 270 ml olive oil, 7.2 ml linseed oil (MIX) as described by van Zwieten *et al.*^31^ In summary: the oil mixture was oxidized over 7 days at a temperature of 90 °C while being continuously stirred. After cooling to room temperature, 20 g oxidised oil was combined with a water-based solution containing 1 wt% Pluronic F-108 (Sigma Aldrich), 2 wt% alginic acid up to a volume of 450 ml. The oxidised oil plus solution were then ground for 10 min using a mixer. The resulting suspension with oil chunks and an added 100 ml of a 1% CaCl_2_ solution was ground further at 15 000 rpm for 15 min to create a solution with adhesive droplets using a Magic Lab (IKA) commercial mixer with gap size of the cone mill of the grinder being 0.318 mm. The final sprayable MIX solutions consisted of 81.6 wt% tap water, 16.4 wt% oxidized MIX oil droplets, 0.2 wt% F-108, of 1.6 wt% alginate and 0.2 wt% CaCl_2_. The density of MIX droplets in the solution with a size > 0.01 mm^2^ was on average around 16 per ml, while mean (±SE) droplet area was 0.83 ± 0.04 mm^2^. After application, the droplets typically exhibit a pull-off force of up to 2.5 mN.^31^ A control solution containing all compounds except adhesive MIX droplets was also made (hereafter referred to as ‘Solution’).

### Insects

A colony of *Frankliniella occidentalis* (obtained from a commercial glasshouse in the Netherlands) was maintained in plastic cages (60 × 60 × 40 cm) at Leiden University for over 15 years on *Chrysanthemum* cut flowers (cv. Baltica Yellow) as described by Bierman *et al.*^29^ Climate room conditions were set at 25 °C, 60% RH (70% inside cages) and a 16–8 light–dark photoperiod (fluorescent TL-light). Each cage contained five vases with *Chrysanthemum* cut flowers. Every two days, the oldest flowers were replaced.

### Plants and growth conditions

One hundred unrooted *Chrysanthemum* × *morifolium* cuttings (cv. Baltica White), obtained from Deliflor B.V. (Maasdijk, The Netherlands) were treated with rooting powder (Chyzoteck beige 0.4%), planted in peat blocks (4 × 4 × 3 cm) and covered with a transparent plastic sheet for 10 days in a climate room at 20 °C, 70% RH and a (long day) 16–8 light–dark photoperiod (fluorescent TL-light, 15 340 lm m^−2^). Cuttings were then transplanted to 2.2 L plastic pots filled with a mixture of 3/1 (vol/vol) autoclaved potting soil to vermiculite, with 2.5 g L^−1^ osmocote fertilizer pellets. Climate room temperature was set to 25 °C during day, 23 °C during night. Watering was done every other day based on need.

### Plant assay with MIX

Eighty undamaged, 25 day-old *Chrysanthemum* plants (17.1 cm [SD: 0.6] shoot length, with 10 unfolded leaves and several smaller budding leaves) were divided into eight groups of ten plants. Two groups of ten plants were randomly allocated to one of four spray treatments: (per plant) 20 ml water, 20 ml Solution (F108 + alginate + water), 20 ml full MIX sample, or 40 ml full MIX sample (2 × 20 ml with a 30 min. drying step between spray events). Spraying was done under 4 bar pressure from 20 cm above plants using a Birchmeier Spray Matic 2S (DCM Nederland B.V., Nieuw-Vennep, the Netherlands) which had been modified to attach a Teejet 2502-SS nozzle (Teejet Technologies Northwest Europe, Schorndorf, Germany) (Fig. S1). After spraying and drying, the number of adhesive droplets per leaf and percentage coverage were estimated by taking pictures of the sprayed leaves of five plants per treatment under blue UV-light (Nightsea Royal blue) (Fig. S2a–c). Plants were then placed on felt circles located on plastic dishes (25 cm diameter) and covered with thrips-proof cages as in earlier work.^32^ The felt circles were kept moist during the experiment. Per spray treatment, one group of ten plants received 20 adult *F. occidentalis*, introduced at the base of the plant. Thrips cages with plants were placed in the climate room in ten blocks with eight plants (one plant per treatment combination). After 25 days with thrips, the number of thrips caught in adhesive droplets and the number of thrips larvae on the plant were counted and thrips damage and necrotic damage (mm^2^) were scored for each leaf on the adaxial and abaxial side. The number of leaves with growth malformations, shoot length (cm) and the total number of unfolded leaves were also recorded. Then, a stack of sprayed older, fully unfolded leaves: leaf number 3 to 9 (counted from the bottom up) and a stack of newly grown (unsprayed) leaves: leaf number 5 to 11 (counted from the top down, generally leaf 12 to 18 or 13 to 19 as counted from the bottom) of five plants per treatment were sampled for HS GC-MS and ^1^H NMR analyses between 7 to 9 pm. Leaves were flash frozen in liquid nitrogen and stored at −80 °C until freeze drying. After freeze drying, leaves were ground into a fine powder and total shoot dry weight was determined as described by Bierman *et al.*^32^

### Analysis of plant growth and thrips performance

Assumptions for normality and homogeneity of variance were tested using Shapiro–Wilk tests and Levene's tests. *P* values < 0.05 were considered significant. Total shoot length, dry weight, and necrosis damage were analyzed with two-way ANOVAs with spray treatment and thrips infestation as factors. Total thrips damage was analyzed for sprayed leaves (1–10) and newly grown leaves (11 and above) separately using one-way ANOVAs. For plants with thrips, differences between treatments in the number of total thrips larvae on a plant and the number of young leaves with malformed growth were analyzed using one-way ANOVAs. Significant one-way ANOVAs were followed up by pairwise comparisons using Tukey tests.

### ^1^H NMR and HS-GC-MS analysis and multivariate data analysis

Preparation, extraction, and data acquisition of the ^1^H NMR and GC-MS samples was done using the same procedures and using the same equipment as in earlier work.^32^ For ^1^H NMR, a large part of this workflow was sourced from the protocol by Kim *et al.*^36^ For each treatment, five replicates were done per sample. 30 mg freeze dried leaf material was used for ^1^H NMR and 100 mg material was used for HS-GC-MS. The obtained ^1^H NMR and HS-GC-MS data were analyzed *via* SIMCA P (version 18.0.1, Sartorius) using multivariate statistical models, specifically principal component analysis (PCA) followed by orthogonal partial least squares discriminant analysis (OPLS-DA) modeling. Pareto-scaling was used for all data. Variance (*R*^2^) and cross-validated variance (*Q*^2^) values (100 permutations) were used to validate the predictive ability of the OPLS-DA models. Through this approach, several key factors influencing the metabolome of *Chrysanthemum* leaves were identified. For the OPLS-DA models, first the metabolomic differences between local, sprayed leaves and systemic, younger leaves were investigated. Second, the samples were categorized based on treatments: water, Solution, 20 ml MIX, and 40 ml MIX, with treatment intensity quantitatively represented as 0 (water and Solution), 1 (MIX20), and 2 (MIX40). Third, the effect of thrips infestation was investigated, with samples classified as either control (non-infected) or thrips infected. Based on this approach, additional OPLS-DA models featuring subsets of the data and S-plots were used to assess which signals were behind the observed separations. Since this approach was untargeted, individual metabolites belonging to significant ^1^H NMR and HS-GC-MS signals were later identified using the procedures described by Liang *et al.*^37^ and Wolfender *et al.*^38^

## Results

### Thrips performance and plant growth

Coverage with adhesive droplets was estimated at 8.16 (SE: 0.73) droplets (>0.5 mm^2^) per leaf or 1.67% (SE: 0.11) of leaf area for 20 ml MIX sprayed plants, and 19.56 (SE: 1.15) droplets per leaf or 4.81% (SE 0.27) of leaf area for 40 ml MIX sprayed plants (Fig. 1a and b). Among all plants, only one thrips individual was found trapped in adhesive droplets present on the *Chrysanthemum* leaves (Fig. 1c). Total thrips damage per plant was lower in the MIX 20 ml treatment than in the water treatment, on both the sprayed leaves (*F*_3,36_ = 3.06, *P* = 0.04; Fig. 2a) and on the unsprayed newly grown leaves (*F*_3,36_ = 5.56, *P* < 0.01; Fig. 2b), while no differences in total thrips damage were found between the water treatment and the Solution or MIX 40 ml treatment. However, comparing the damage patterns in higher resolution per individual leaf revealed that the 40 ml MIX treatment showed a similar damage pattern to the 20 ml MIX treatment, especially in the middle region of the plant around leaf number 9 to 13 (the youngest leaf tissue at the time of spraying), suggesting that also the 40 ml MIX application does affect thrips feeding behavior (Fig. 2c–e). Malformed growth of young leaves was only observed in plants with thrips and not in control plants (Fig. S3). Spray treatments were not effective to reduce the number of leaves with malformed growth on plants infested with thrips (*F*_3,36_ = 0.44, *P* = 0.73). Thrips larvae were located mostly in the top of the plant, hidden between the youngest budded leaves. More larvae were found on plants that had been sprayed with water than on plants that had been sprayed with 20 ml MIX or 40 ml MIX sample (*F*_3,36_ = 4.34, *P* = 0.01, Fig. 2f). Plants with thrips had a lower mean shoot length (42.9 cm, SE: 0.2) (*F*_1, 72_ = 63.21, *P* < 0.001) than plants without thrips (45.3 cm, SE: 0.2). Spray treatment had no visible effect on shoot length (*F*_3,72_ = 1.50, *P* = 0.22). There was no significant interaction between spray treatment and thrips infestation (*F*_3,72_ = 0.90, *P* = 0.44). Regarding mean shoot dry weight (Fig. 2g), there was also no interaction between spray treatment and thrips infestation (*F*_3,72_ = 0.19, *P* = 0.90). Plants with thrips had less mean shoot dry weight (3.96 g, SE: 0.12) than plants without thrips (4.25 g, SE: 0.06) (*F*_1,72_ = 4.50, *P* = 0.04) and there was no significant effect of spray treatment (*F*_3,72_ = 0.23, *P* = 0.88). Spraying plants with the full MIX sample caused phytotoxic damage (Fig. S4), which could be seen as necrotic brown spots where oily droplets contacted the leaf surface. Spraying only the Solution did not produce such brown spots. Plants sprayed with 40 ml MIX sample exhibited more phytotoxic damage (100.0 mm^2^, SE: 8.3) than plants sprayed with 20 ml MIX (30.8 mm^2^, SE: 3.2) (*F*_3,72_ = 106.58, *P* < 0.001). Thrips infestation had no significant effect on phytotoxic damage (*F*_1,72_ = 0.001, *P* = 0.97), nor was there an interaction between thrips infestation and spray treatment (*F*_3, 72_ = 0.18, *P* = 0.91).

**Fig. 1:**
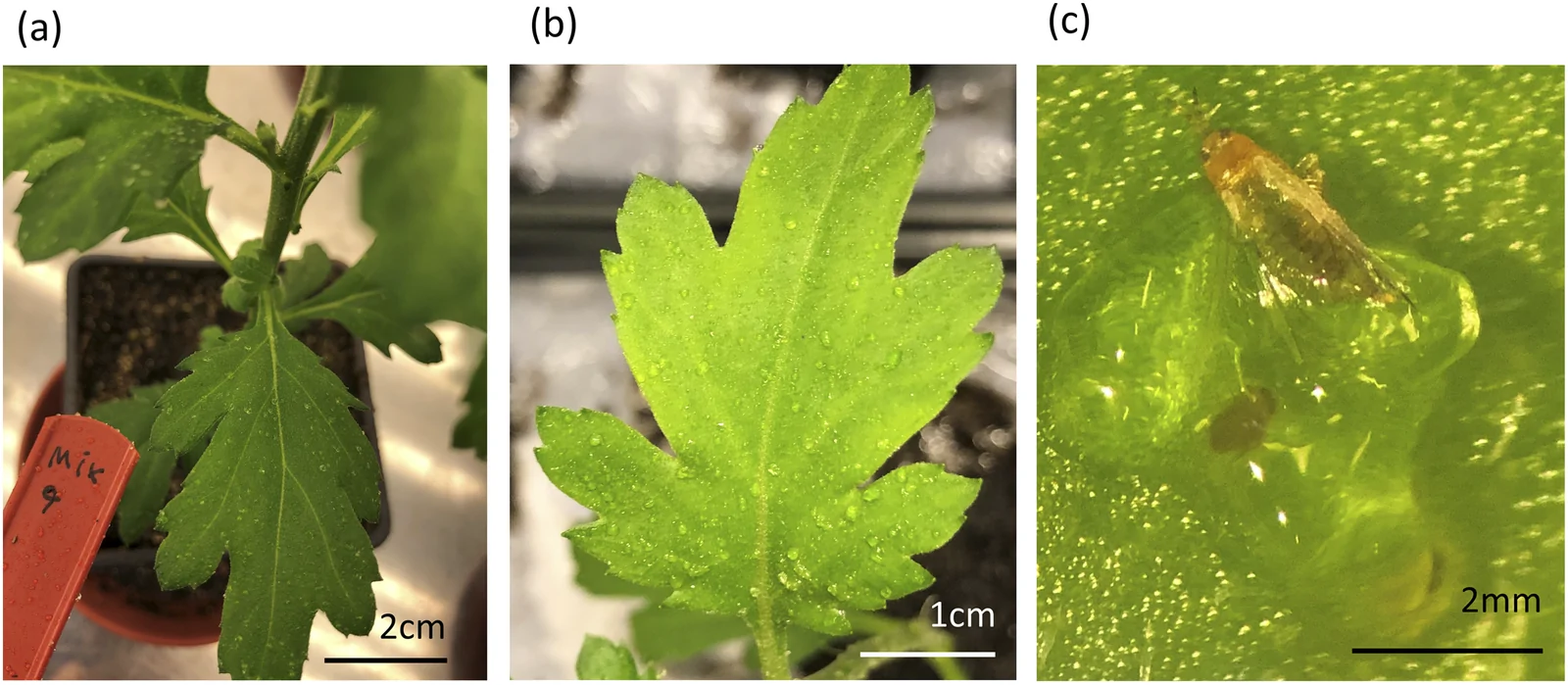
Example of coverage with MIX on the 4^th^ leaf from the bottom (a), example of MIX coverage on the 10^th^ leaf from the bottom (b), the only thrips found stuck in a MIX droplet (c).

**Fig. 2:**
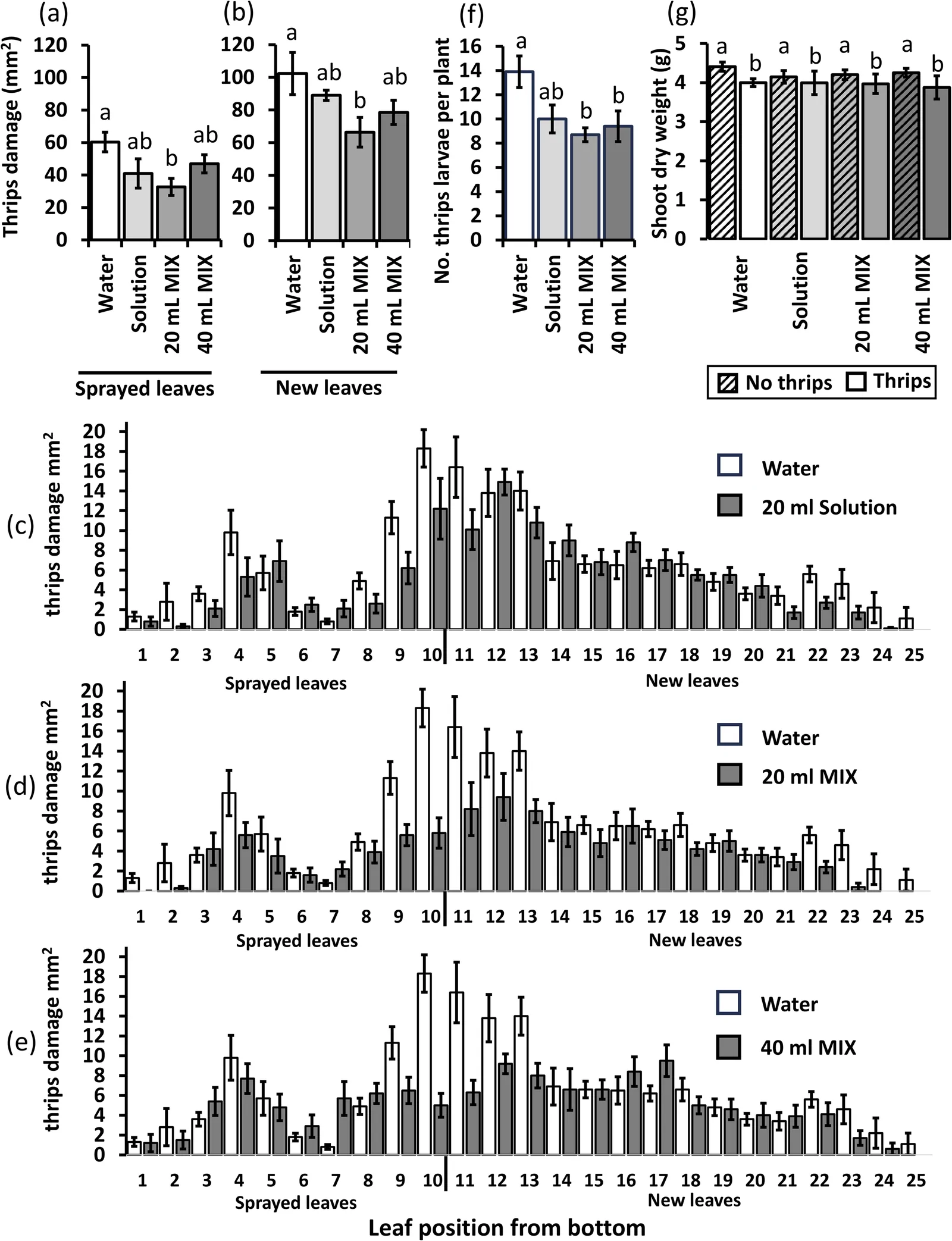
Plant assay with MIX (*n* = 10): total mean (±1 SE) thrips damage (mm^2^) on sprayed leaves (a) and newly grown leaves (b) of thrips infested *Chrysanthemum* plants sprayed with several treatments: 20 ml water, 20 ml Solution (water with alginate, F-108 and CaCl_2_), 20 ml MIX (Solution + MIX oil droplets) and 40 ml MIX. Mean thrips damage (mm^2^) per leaf position (leaf number 1 to 25, counted from the bottom) of each spray treatment compared to the water control (c–e). Leaves 1–10 (fully unfolded) were sprayed. Leaves 11–25 (young leaves and buds at the time of spraying) were considered newly grown. Mean number of thrips larvae found on plants sprayed with different treatments (f). Mean total shoot dry weight (grams) of sprayed plants that were infested with 20 thrips or without thrips (g). Different letters indicate significant differences at *P* < 0.05 between spray treatments as found by (a, b and f) one-way ANOVAs and Tukey *post hoc* tests or (g) two-way ANOVA.

### ^1^H NMR and HS-GC-MS results

As illustrated in Fig. 3, the PCA score plots revealed that the primary factor influencing the metabolome of *Chrysanthemum* leaves was leaf location. Older, sprayed (local) leaves were clearly separated from unsprayed, newly grown (systemic) leaves in the ^1^H NMR and HS-GC-MS data. Interestingly, in the ^1^H NMR dataset (Fig. 3a), the local leaves exhibited distinct metabolic differences between water/Solution sprayed leaves and leaves sprayed with 20 ml Mix and 40 ml MIX, whereas the systemic leaves did not display such differentiation. Conversely, volatile compounds detected *via* HS-GC-MS did not show a clear separation between spray treatments (Fig. 3b).

**Fig. 3:**
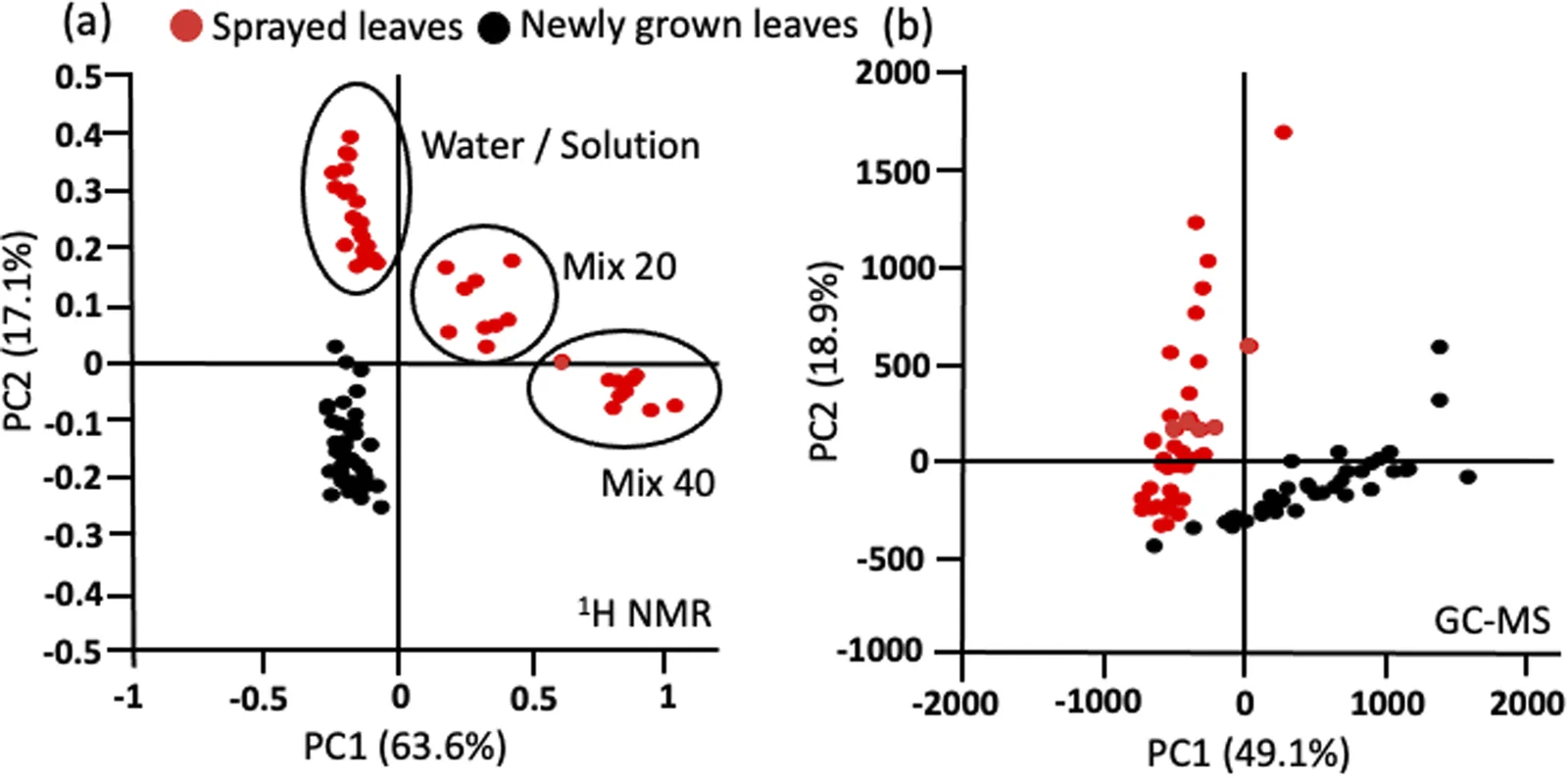
Score plot of principal component 1 (PC1) *vs.* PC2 of principal component analysis (PCA) of ^1^H NMR data (a) and HS-GC-MS data (b) of the leaves of *Chrysanthemum* plants that were sprayed with several treatments: 20 ml water, 20 ml Solution (water with alginate, F-108 and CaCl_2_), 20 ml MIX (Solution + MIX oil droplets) and 40 ml MIX (*n* = 5). The red dots are the older, sprayed leaves and the black dots are the unsprayed, newly grown leaves. Significantly different groups of datapoints are indicated using ellipses.

Following PCA, OPLS or OPLS-discriminant analysis (OPLS-DA) was applied in a supervised manner to examine the effects of each experimental factor (leaf location, spray treatment, and thrips infestation) in more detail. As shown in Fig. 4a and b the OPLS-DA results aligned with the PCA findings, again demonstrating a clear distinction between the sprayed, local and unsprayed, systemic leaves in the ^1^H NMR and HS-GC-MS datasets. A separation between spray treatments was also again visible in the OPLS-DA plot of the ^1^H NMR dataset.

**Fig. 4:**
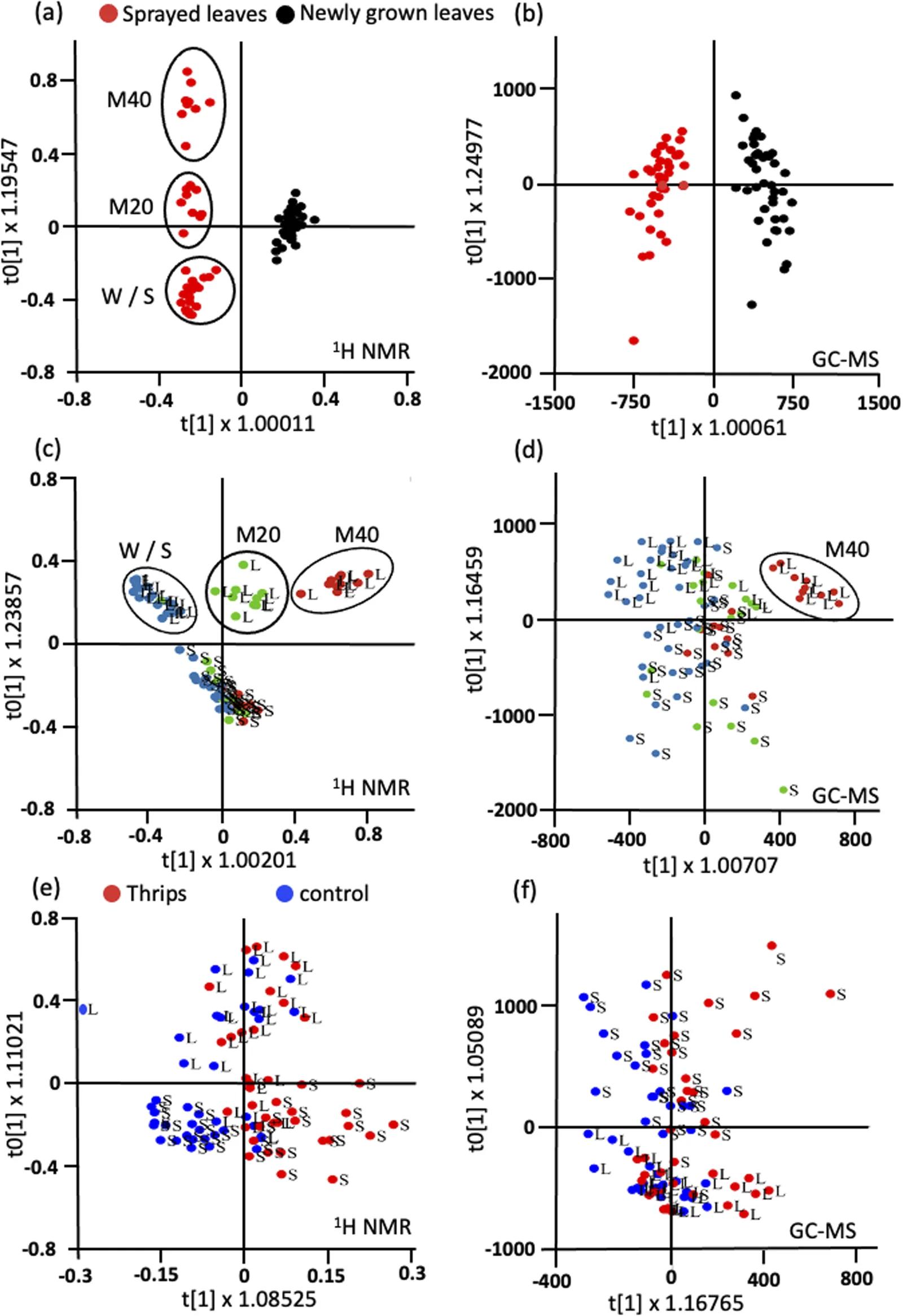
Score plots of predictive component *t*[1] and an orthogonal component *t*0[1] of orthogonal partial least square (discriminant analysis) modeling of ^1^H NMR data and HS-GC-MS data using class variables of local and systemic leaves (a and b), the applied dose of MIX (W/S: water and Solution, M20: 20 ml MIX, M40: 40 ml MIX) (c and d), and control and thrips infected leaves (e and f) (*n* = 5). The letters in (c–f) are the sprayed, local (L), and newly grown, systemic (S) leaves, respectively.

Since the water and Solution treated leaf samples remained grouped together in the PCA and OPLS-DA plots, the effect of spray treatment was further analyzed using a quantitative variable for the quantity of MIX oil present. This variable ranged from ‘0’ (water/Solution) to ‘2’ (40 ml MIX sample), with ‘1’ representing the 20 ml MIX treatment. Using this approach, metabolic differences were again found to be more pronounced in the local leaves than in the systemic leaves, especially in the ^1^H NMR dataset, while in the HS-GC-MS data a separation was now also visible between spray treatments in the local leaves (Fig. 4c and d).

Compared to the other factors, the metabolic differences associated with thrips infestation (no thrips *vs.* thrips) were less pronounced (Fig. 4e and f). The ^1^H NMR data exhibited a marginal separation between the leaves of thrips infested and control plants with a low *Q*^2^ value (0.317). Interestingly, in the ^1^H NMR dataset, the systemic leaves (where the thrips larvae were mostly located during the experiment) showed a more distinct separation than the local leaves. The same patterns were not observed in the HS-GC-MS data as the separation was not validated (*Q*^2^ value = 0.00417).

For both the ^1^H NMR and HS-GC-MS datasets, the contribution of specific metabolites to the observed separations for each factor were subsequently examined using S-plots based on the OPLS-DA models with all samples or on OPLS-DA models featuring subsets of the data. For the ^1^H NMR data, the local leaves showed higher levels of fatty acids, lipids, and glutamic acid compared to systemic leaves (Fig. S5a). In addition, clear differences were also observed in the characteristic region for phenylpropanoids (H-8 resonances), where various phenylpropanoids were more abundant in local leaves (Fig. S5b). Among these phenylpropanoids, 3,5-dicaffeoylquinic acid was identified as a predominant compound. In contrast, the systemic leaves exhibited higher levels of phytic acid and *myo*-inositol than local leaves. For the HS-GC-MS data, the levels of several volatile compounds were found to differ between the local and systemic leaves (Fig. S5c). 2,6-Dimethyl-3,5-heptadiene was more abundant in systemic leaves but was only detected in leaves that were not sprayed with MIX. In contrast, the local leaves, particularly those sprayed with MIX, exhibited higher levels of 1-octen-3-ol (eucalyptol) and 6-isopropylidenebicyclo[3.1.0]hexane.

Since the OPLS models of the full data (Fig. 4c and d) indicated that MIX application predominantly influenced the local leaves, additional OPLS modeling was done using only the local leaf data. The resulting OPLS-DA plot again showed a clear separation between spray treatments with 20 ml and 40 ml MIX and the cluster of the Solution/water treatments (Fig. S6a). For the ^1^H NMR data, the S-plots indicated that phospholipid content in the local leaves increased with higher applied doses of MIX, while unsaturated fatty acid levels decreased (Fig. S6b and c). For the HS-GC-MS data, distinct metabolite changes were also visible depending on the applied dose of MIX. Specifically, octanal, nonanal, and eucalyptol were more abundant in local leaves treated with higher MIX concentrations, whereas 2,6-dimethyl-3,5-heptadiene, chrysanthenone, and aromadendrene were less prevalent.

Separate OPLS-DA modeling of the effect of thrips infestation on the ^1^H NMR profile of the local (Fig. 5a) and systemic (Fig. 5b) leaves led to an improved model for the systemic leaves with a *Q*^2^ value of 0.828, whereas for local leaves no separation was visible and the *Q*^2^ remained negative at −0.144. The separation in the systemic leaves was primarily driven by higher levels of phenolics in the leaves of thrips infested plants, particularly phenylpropanoids and choline analogs. In contrast, unsaturated fatty acids were more abundant in systemic leaves of the no-thrips treatment. Separate OPLS-DA modelling of the local and systemic leaf HS-GC-MS data (Fig. 5c and d) did not lead to a model with clear separations, although the *Q*^2^ value for the systemic leaves was slightly improved, increasing from 0.00417 to 0.144.

**Fig. 5:**
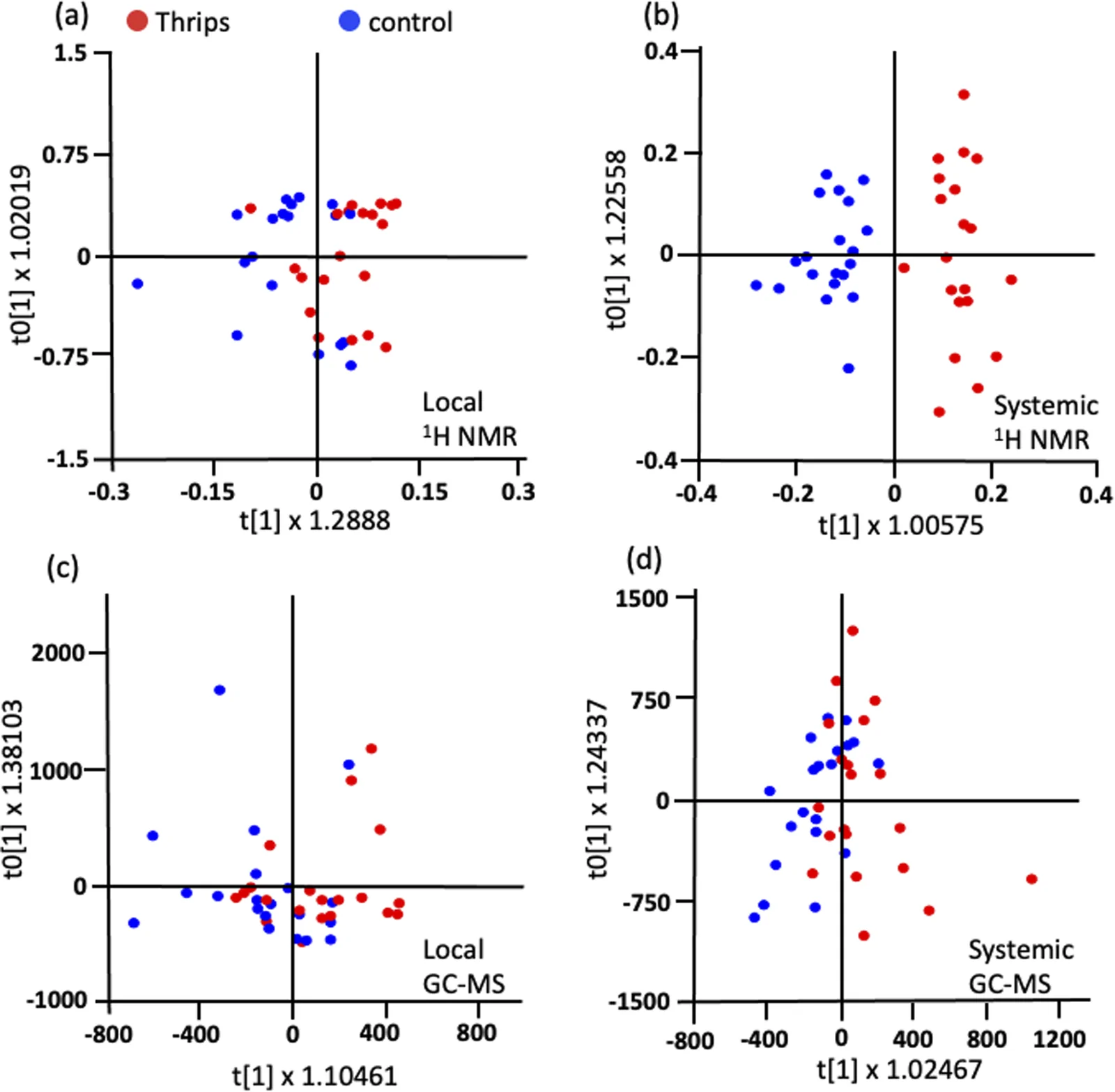
Score plots of predictive component *t*[1] and an orthogonal component *t*0[1] of orthogonal partial least square (discriminant analysis) models of ^1^H NMR data (*n* = 5) of only sprayed, local leaves (a), ^1^H NMR data of only newly grown, systemic leaves (b), head space gas chromatography mass spectrometry data of sprayed, local leaves (c), and headspace gas chromatography data of newly grown, systemic leaves (d). Within OPLS-DA models, samples were classified in control (blue dots) and thrips infected (red dots).

## Discussion

In search of new sustainable pest control methods, we tested whether solutions containing natural chemicals and adhesive droplets made from plant-based oils could make *Chrysanthemum* plants less susceptible to thrips *via* direct effects on the pest and *via* changes in the plant metabolome. We found that spraying solutions containing adhesive droplets made from a mixture of plant-based oils (MIX) was not effective to physically trap thrips on plant surfaces and did not significantly reduce the number of leaves with malformations. However, spraying did reduce total thrips damage and reproduction, with the damage reductions being visible especially on the middle leaves of the shoot. This suggests that the mechanism of thrips control achieved with this method is not based on physical adherence. Furthermore, the MIX spray affected the metabolome of *Chrysanthemum*. The effect of MIX spraying was the clearest in the ^1^H NMR and HS-GC-MS profiles of the sprayed, local leaves, whereas systemic effects in newly grown parts of the plants were not observed. Local leaves treated with 20 ml MIX and 40 ml MIX were clearly separated and showed higher contents of phospholipids, octanal, nonanal, and eucalyptol compared to leaves sprayed with water or with the control Solution, which had relatively higher levels of unsaturated fatty acids, 2,6-dimethyl-3,5-heptadiene, chrysanthenone, and aromadendrene. The metabolomic effects of MIX spraying did not seem to be influenced significantly by thrips presence, which by itself caused a local increase in the content of phenolics in the newly grown leaves, especially phenylpropanoids and choline analogs, while reducing unsaturated fatty acid levels. Overall, sprayed leaves featured higher levels of fatty acids, lipids, glutamic acid, phenylpropanoids like 3,5-dicaffeoylquinic acid (isochlorogenic acid A), eucalyptol, and 6-isopropylidenebicyclo [3.1.0] hexane. Newly grown, younger leaves showed higher levels of phytic acid, *myo*-inositol and (only for the water/Solution sprayed leaves) 2,6-dimethyl-3,5-heptadiene.

These findings agree with other studies that have shown that the application of plant derived compounds can directly repel, poison, or otherwise negatively affect arthropods^14,15,39–42^ and with studies that show that such compounds can induce plant chemical defenses against pests and diseases.^43,44^ Our results also match with observations from earlier studies where similar solutions with adhesive rice germ oil (RGO) droplets^32^ or jasmonic acid (JA) solutions^45^ were applied to *Chrysanthemum* plants. In these studies, spraying plants also reduced total thrips damage and resulted in changes in the levels of several metabolites in locally sprayed *Chrysanthemum* leaves. Similar to the current study where MIX was applied, the metabolomic changes due to product application in the previous study with RGO also seemed little affected by thrips presence.^32^ Interestingly however, both the application of RGO,^32^ JA,^45^ or MIX, and thrips feeding seemed to result in similar increases or decreases in several compounds (*e.g.* higher phenylpropanoids and flavonoids and lower chrysanthenone concentrations). These similarities suggest that the RGO and MIX formulations and thrips feeding both induced a more general metabolomic response. This response was likely mediated *via* stimulation of the jasmonic acid/salicylic acid pathway, which is known to be induced by thrips,^46^ and possibly *via* other pathways, which are thought to be interconnected and intimately involved in shaping the local and systemic defense responses of plants to simultaneous abiotic and biotic stresses.^47,48^ General patterns inferred from numerous other studies on the anti-herbivore, phytotoxic, and general plant defense inducing effects of plant-based oils and other chemicals further support this conclusion and provide abundant evidence that systemic effects of natural product application and local effects of herbivore feeding are also common.^49–52^ As such there seems to be a solid foundation for the application of plant-based oils to make plants more resilient against agricultural pests.

In our study, we did not observe strong systemic metabolomic effects after MIX application. This could be due to the location where we applied our compound, where we sampled the plant, and the time of sampling. The induction of older plant tissues is generally observed to lead to a weaker systemic response than when young tissues are stimulated directly.^53–55^ When we sprayed plants on leaves 1 to 10 (counted from the bottom up), interestingly leaf 9 to 13 showed the largest differences in thrips damage (Fig. 2c–e), suggesting that metabolomic differences may have been the strongest in the leaves closest to the inoculation point. Meanwhile, we sampled locally sprayed leaf 3–9 and systemic leaf number 5 to 11 from the top down (generally leaf 12–18 or 13–19 from the bottom). Hence, in our samples, we may have missed the region closest to the inoculation where natural product application caused the largest effects in the leaves. Further, the response of plants to compound application can be very time sensitive.^56^ Plants can initiate their defensive responses within minutes^57^ and these changes typically last for several hours to a few days, after which levels may decline but can again be re-induced.^58^ In the current study we sampled leaves after 25 days since our aim was to quantify whether spraying could induce strong, lasting metabolomic effects in newly grown leaves. It is possible that the systemic metabolomic effects of MIX application may have already disappeared at the 25 day timepoint. Similar to the studies mentioned before, our data suggests that newly formed tissues may be less affected by the application of plant-based oils than tissue present during inoculation, and that the effects of spraying with plant-based oils on plant metabolomic responses may decline over time.

Regarding the effectiveness of the MIX sample, several of its constituents have been found in other studies to affect pests and diseases and the plant metabolome. Sunflower oil application can repel horn flies from cattle^59^ and can lower powdery mildew incidence in tomato *via* direct effects, but not *via* induction of resistance pathways.^60^ Linseed oil application lowered population sizes of cotton jassid (*Amrasca biguttula*), whitefly (*Bemisia tabaci*) and onion thrips (*Thrips tabaci*) in cotton.^61^ Olive oil application to bean plants has been shown to reduce damage by ozone by inducing the closure of stomata.^62^ Interestingly, the higher dose of MIX application in our study did not lead to stronger reductions in thrips performance, suggesting that the oils used in the MIX formulation are not severely toxic to *F. occidentalis*. Furthermore, the control Solution containing the F108 surfactant, alginate, and CaCl_2_ without the MIX oil component was not found to induce metabolomic effects and seemed to have little activity against thrips, failing to significantly reduce damage or the number of larvae on the plant. This finding disagrees with the previous study with RGO where such control solutions did reduce thrips damage substantially.^32^ Several other studies also report clear effects of the application of CaCl_2_, alginate, and surfactants on herbivory and the plant metabolome. For example, Affeld *et al.*^63^ reported anti-arthropod effects of F-108, Saberi Riseh *et al.*^64^ showed that alginate application induces plant defensive pathways against diseases, while Zeng *et al.*^65^ and Qiu *et al.*^66^ observed that application of CaCl_2_ on kidney bean induced the activities of lipoxygenase (LOX), phenylalanine ammonia lyase (PAL), and β-1,3-glucanase and caused changes in the content of tannins, flavonoids, total phenol, alkaloids, and lignin in leaves and reduced *F. occidentalis* fitness.

Considering the observed alterations in the *Chrysanthemum* metabolome, several of the metabolites that were affected by MIX application are known to be related to plant responses to abiotic and biotic stress. Phenylpropanoids such as chlorogenic acid have been linked to thrips resistance^67^ and generally fulfill a wide range of functions in plant defense.^68,69^ Phenols are similarly known to be involved in defense against herbivores, microbes, and other plants.^70^ Meanwhile, choline and changes in fatty acid and lipid content, are involved in abiotic and biotic stress tolerance.^71,72^ Aldehydes such as octanal and nonanal are also commonly induced following herbivore or abiotic stress.^73^ Eucalyptol and chrysanthenone, both major volatile constituents of *Chrysanthemum*, are generally known to be repellent or toxic to arthropods.^74–77^ However, like chrysanthenone, eucalyptol seems attractive to western flower thrips^78,79^ rather than repellent or toxic.^80^ Aromadendrenes, normally repellent to herbivores, have been found to also reduce under cadmium stress.^81^ Glutamic acid plays a key role in plant metabolism signaling including in responses to all kinds of stress.^82^ Phytic acid, among other functions, acts as an anti-feedant for herbivores^83^ while *myo*-inositol acts in diverse pathways, including in the pathway towards phytic acid and in abiotic stress responses.^84^ Further investigations of these compounds and their underlying pathways and genetics will likely be useful to determine the appropriate targets for further resistance breeding.

Using metabolomics techniques such as ^1^H NMR (nuclear magnetic resonance) and headspace GC-MS (gas chromatography - mass spectrometry), thrips resistance in *Chrysanthemum* has also been linked to several other metabolites, including an unsaturated isobutylamide and the phenylpropanoid feruloyl quinic acid.^67,85,86^ In other crops, pyrrolizidine alkaloids, α-tomatine, amino acids such as threonine, citric acid, β-alanine, several terpenes, flavonoids and other phenolic compounds have also been associated with increased resistance against thrips, while higher glucose content was associated with increased thrips feeding.^23,35,55,87–91^ For many other plant compounds, their function in plant resistance against abiotic and biotic stresses remain to be discovered. To fully utilize natural materials for crop protection against various pests, further investigations will be required to understand the intrinsic interactions between natural materials, the plant systemic response to crop protection products and herbivore feeding. Metabolomics techniques have a great potential to aid in these investigations.^92,93^

In conclusion, in this study we show that the application of solutions containing adhesive droplets made from plant-based oils, despite not being an efficient thrips trapping method, is an effective way to make plants less susceptible to western flower thrips. The metabolites we identified could be further investigated for resistance breeding against thrips and other stressors. Furthermore, our findings highlight that the effects of such natural products on the plant metabolome are mostly local and persist over longer time periods following a single application. From an applied perspective, the use of solutions featuring natural chemicals and plant-based oils holds potential for use in agriculture. However, which natural materials are most effective, in what dose, in what way, and how often to use these materials to stimulate different plants maximally on a systemic level to achieve optimal crop protection under different conditions remain questions to be addressed in future studies.

## Author contributions

All authors took part in conceptualization of the work. RvZ and TEK created the adhesive droplet suspensions at Wageningen University. TVB and HPF performed the plant assay under supervision of KV, TMB and YHC at Leiden University. Data analysis was performed by TVB, HPF, SS and YHC. TVB, TMB and YHC created the figures and wrote the first draft of the manuscript. All authors provided feedback on the manuscript, were involved in proofreading and editing, and approved the final version of the manuscript.

## Conflicts of interest

A patent for the method to fabricate solutions with adhesive plant-derived oil droplets has been filed with the European Patent Office, application no. 22202752.6; EP4356732A1, by Wageningen University.

## Supplementary Material

RA-OLF-D6RA07230E-s001

## Data Availability

The datasets belonging to this study can be found on the online repository Zenodo: https://zenodo.org/records/15653050. Supplementary information (SI): regarding methods, results and statistics. See DOI: https://doi.org/10.1039/d6ra07230e.
