# Supplementary material for: Plant-oil-based adhesive formulations induce plant stress responses and enhance resistance of Chrysanthemum to western flower thrips
Source: RSC Adv. 2026 Oct 27;16(49):66699–711. doi: 10.1039/d6ra07230e (PMC13711384; doi:10.1039/d6ra07230e)
Supplement: Supplementary file 1 [file RA-016-D6RA07230E-s001.pdf]

This journal is © The Royal Society of Chemistry

SUPPLEMENTAL INFORMATION

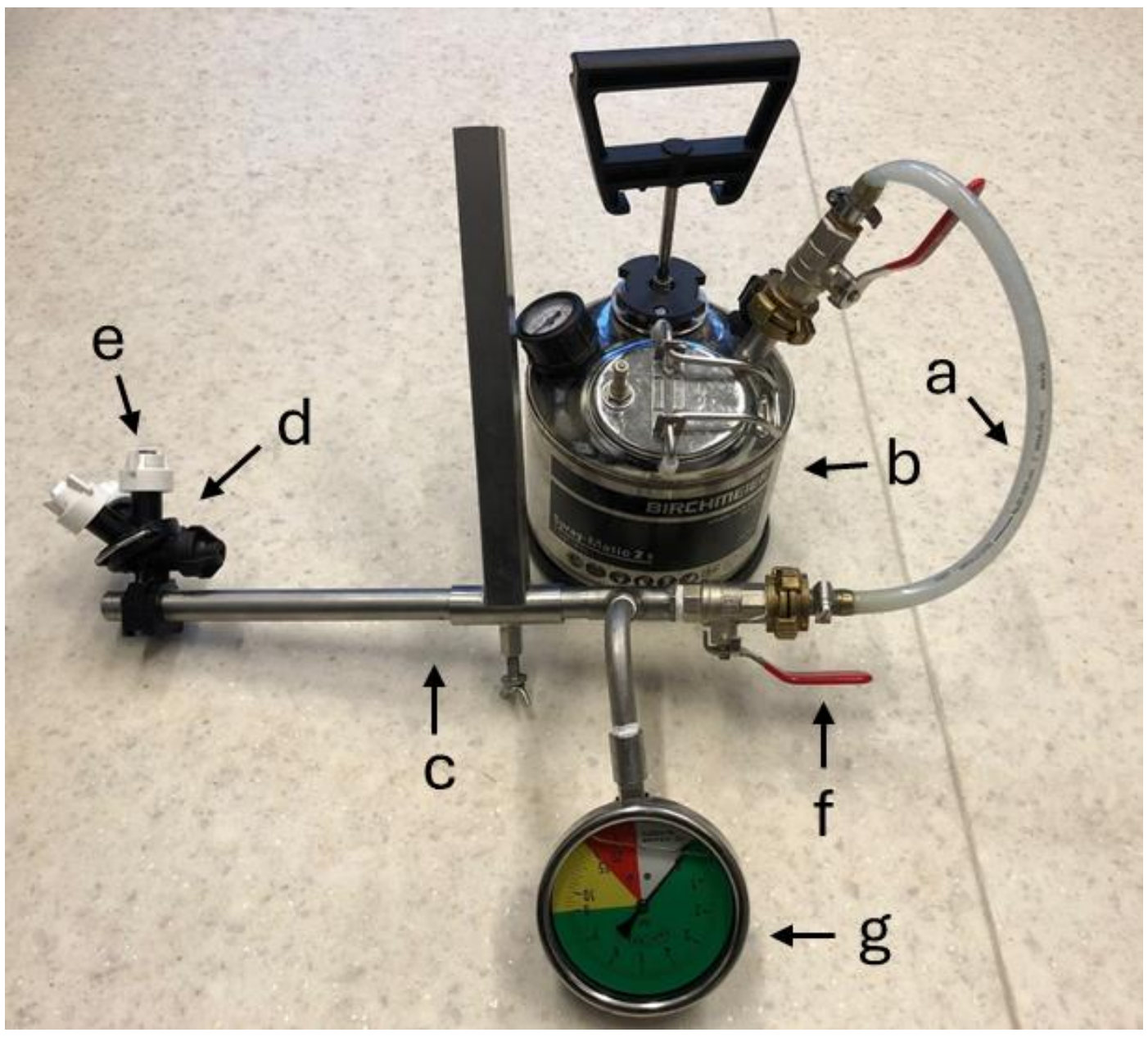

Fig. S1 Modified Birchmeyer Spray Matic 2S (DCM Nederland B.V., Nieuw-Vennep, The Netherlands). A silicone tube (a) is used to connect the tank (b) to a metal tube (c) with connector pieces on the far end (d) to attach the Teejet 2502-SS nozzle (e) (Teejet Technologies Northwest Europe, Schorndorf, Germany). The red valves (f) on the metal tube are used to start and stop spraying while a pressure gauge (g) is used to monitor the amount of spraying pressure in bar

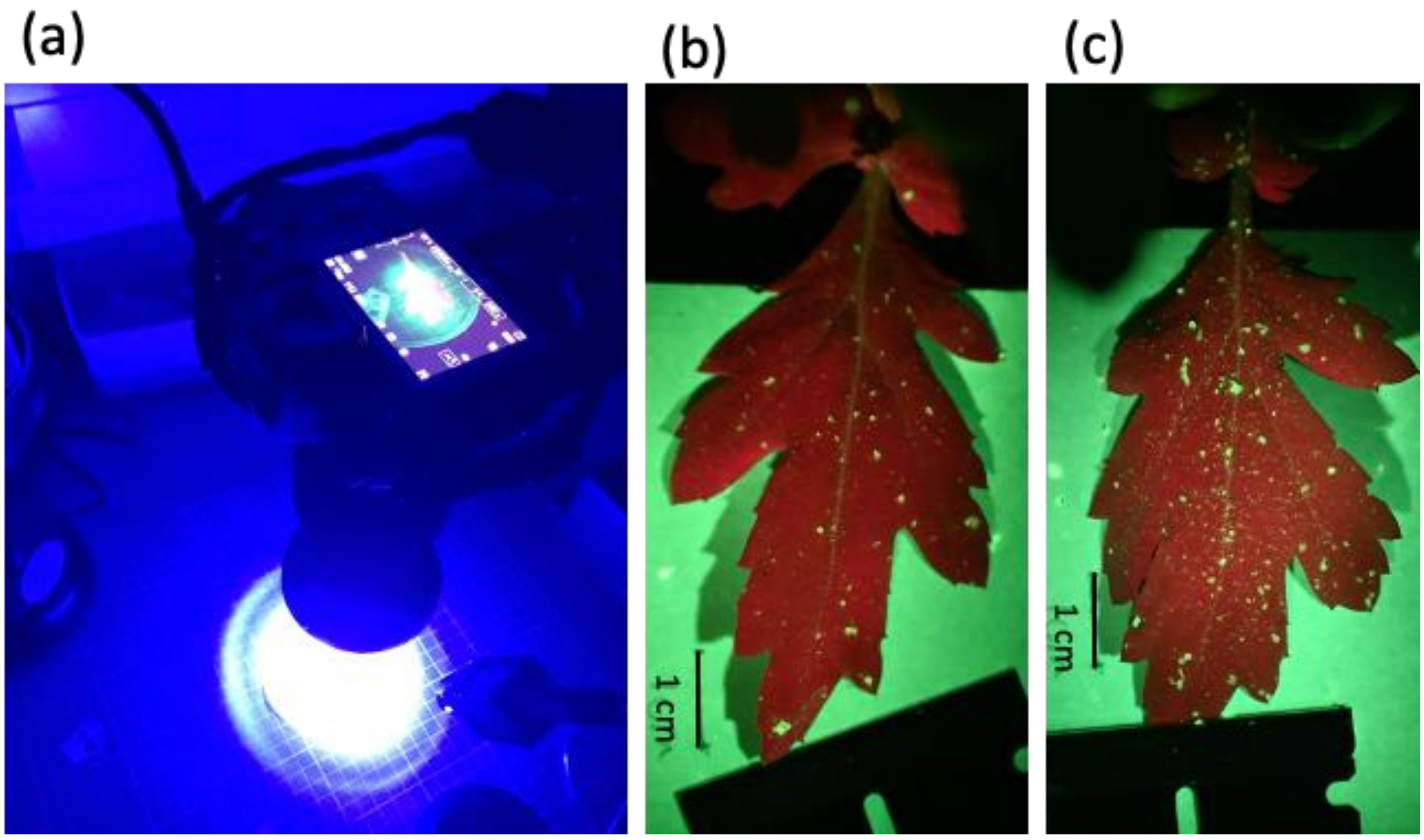

Fig. S2 Camera setup with blue UV ligt (Nightsea^{tm} Royal blue) (a), Example of coverage on the 4th leaf from the bottom of a 20 ml MIX sprayed plant (b) and on the 4th leaf of a 40 ml MIX sprayed plant (c)

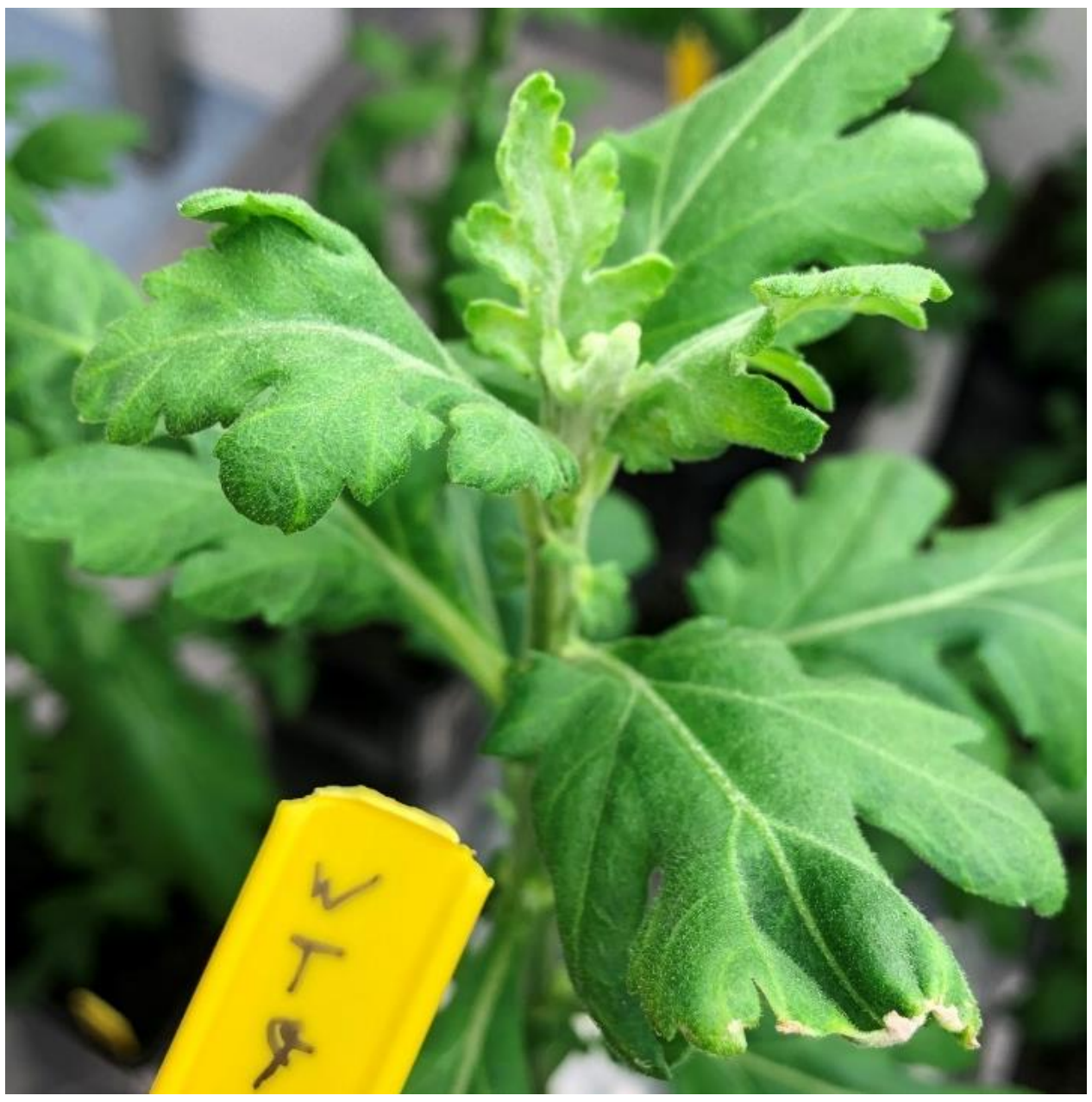

Fig. S3 Malformed growth in the water control treatment of plant assay MIX. The development damage is caused by thrips larvae feeding in the top of the plant and / or by systemic responses of the plant to this feeding

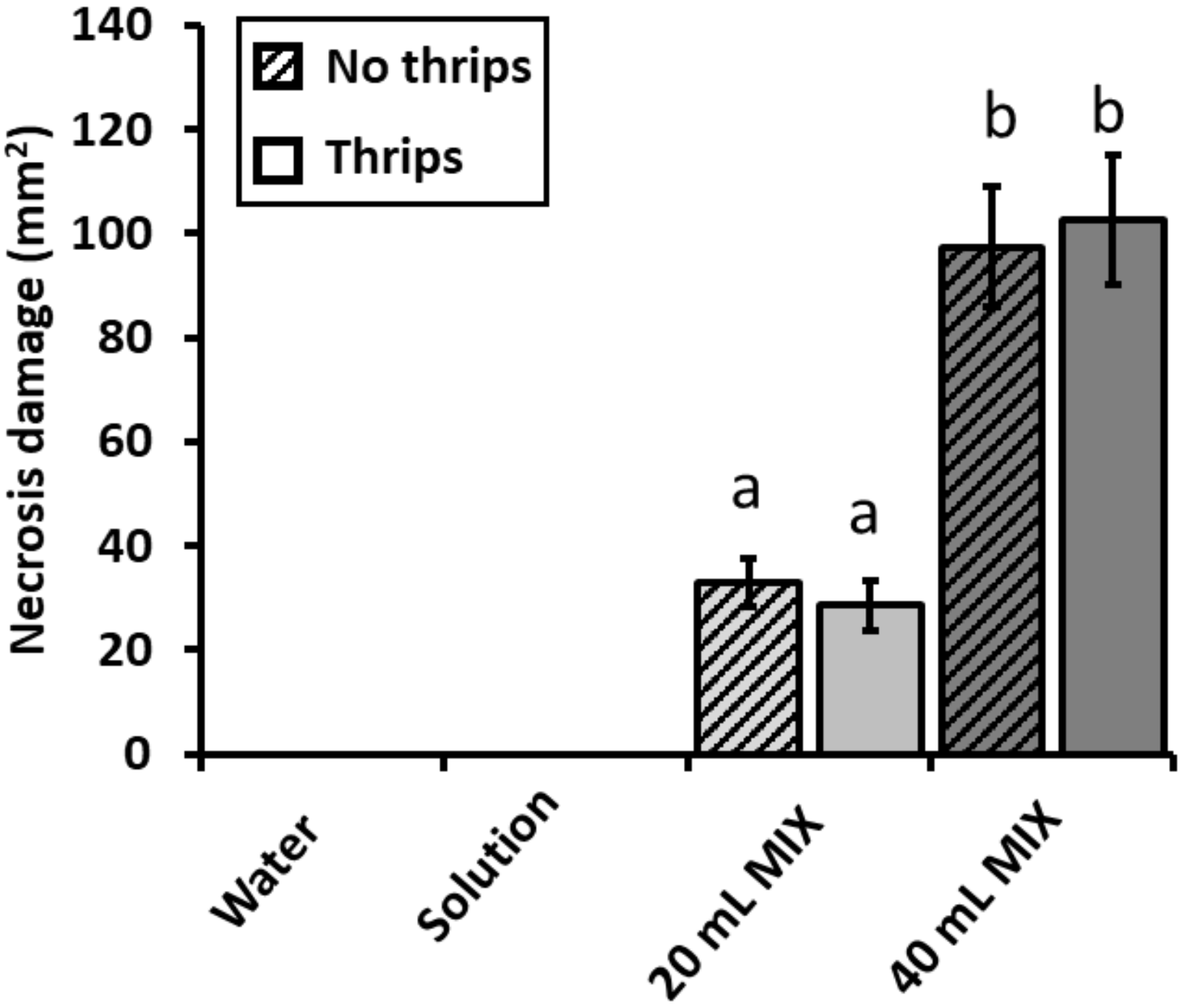

Fig. S4 Plant assay with MIX, mean (± SE) phytotoxic (necrosis) damage in mm^2 of plants that were sprayed with several treatments: water, solution (water with alginate, F-108 and $Cacl_2$), 20 mL solution with adhesive MIX oil droplets and 40 mL solution with MIX droplets and infested with thrips. Letters indicate significant differences as shown by factor analysis via two-way ANOVA, ($P < 0.05$)

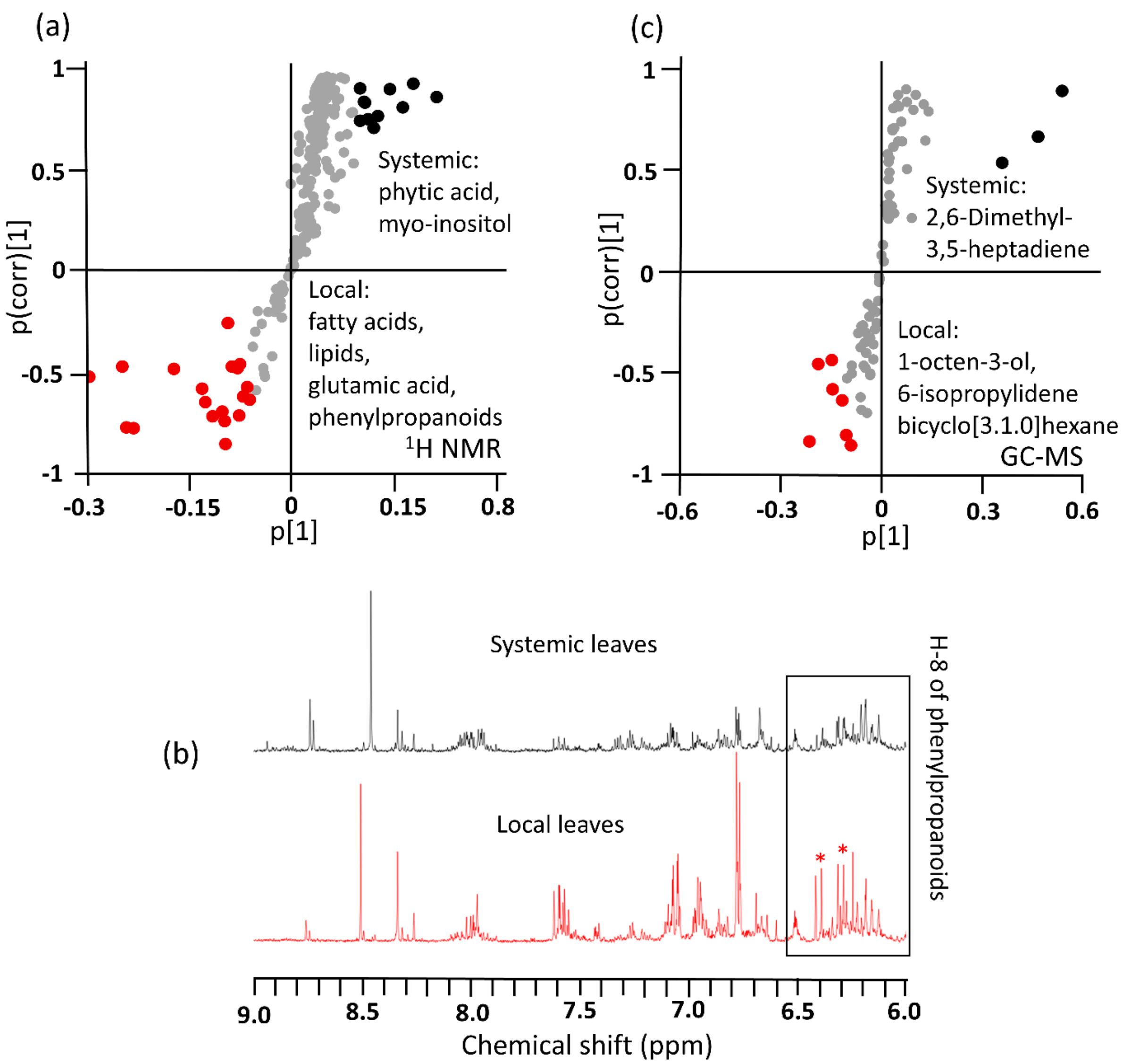

Fig. S5 S-plot of orthogonal partial least square model including all ^{1}H NMR data, classified in local or systemic (a), comparison of ^{1}H NMR spectra in a characteristic phenolic region (δ6.0 – δ9.0) (b), and S-plot of orthogonal partial least square modeling of HS-GC-MS data, classified in local or systemic (c). Asterisk *: H-8 of 3,5-dicaffeoylphenylpropanoids

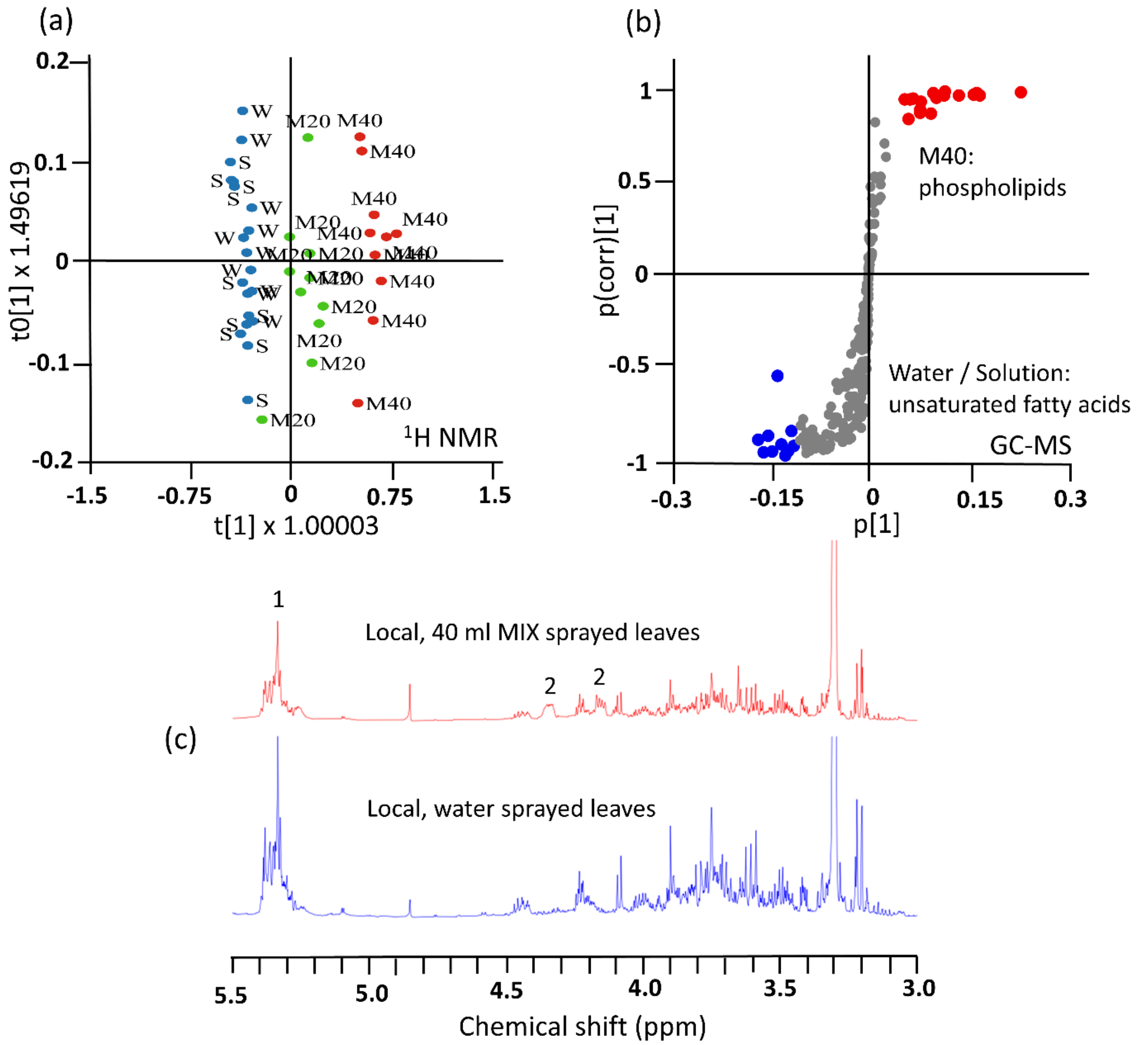

Fig. S6 Score plot (a) and S-plot (b) of orthogonal partial least square (discriminant analysis) models of ¹H NMR dataset of local leaves, classified by spray treatment (blue: Water, "W", Solution "S", green: 20 ml MIX, "M20", red: 40 ml MIX, "M40") (n = 5), and the comparison of ¹H NMR spectra of water / Solution- and 40 ml MIX-sprayed leaves (c) in the range of δ3.0 – δ5.5 "1": ¹H resonance of unsaturated fatty acids, "2": ¹H resonances of phospholipids

SI Table 1 Plant assay with MIX, statistics of ANOVAs

Measured variable and R2	***Source of Variation***	***SS***	***df***	***MS***	***F***	***P***
Total thrips damage on sprayed	Corrected Model	4047.88*	3	1349.29	3.06	0.04
leaves	Intercept	81812.03	1	81812.03	185.64	< 0.01
	spray treatment	4047.88	3	1349.29	3.06	0.04
* R^2 = 0.203, Adj. R2 = 0.137	Error	15865.1	36	440.7		
	Total	101725	40			
Total thrips damage on newly	Corrected Model	0.11*	3	0.04	5.56	< 0.01
grown leaves	Intercept	16.1	1	16.1	2545.5	< 0.001
	spray treatment	0.29	3	0.04	5.56	< 0.01
* R^2 = 0.317, Adj. R2 = 0.260	Error	16.43	36	0.006		
	Total	0.33	40			
Number of thrips larvae per plant	Corrected Model	162.6*	3	54.2	4.34	0.01
	Intercept	4410	1	4410	353.27	< 0.001
* R^2 = 0.266, Adj. R2 = 0.204	spray treatment	162.6	3	54.2	4.34	0.01
	Error	449.4	36	12.48		
	Total	5022	40			
Number of leaves with growth	Corrected Model	1.48*	3	0.49	0.44	0.73
damage due to thrips feeding	Intercept	1677.03	1	1677.03	1490.7	< 0.001
per plant	spray treatment	1.48	3	0.49	0.44	0.73
	Error	40.5	36	1.13		
* R^2 = 0.035, Adj. R2 = -0.045	Total	1719	40			

SI Table 2 Plant assay with MIX, statistics of Two-way ANOVAs

Measured variable and R2	***Source of Variation***	***SS***	***df***	***MS***	***F***	***p***
Chrysanthemum shoot length	Corrected Model	131.05*	7	18.72	10.06	< 0.001
	Intercept	155849.51	1	155849.5	83753.8	< 0.001
* R^2 = 0.494, Adj. R2 = 0.445	spray treatment	8.39	3	2.8	1.5	0.22
	thrips	117.61	1	117.61	63.21	< 0.001
	spray treatment x thrips	5.04	3	1.68	0.9	0.44
	Error	133.98	72	1.86		
	Total	156114.54	80			
Chrysanthemum shoot dry weight	Corrected Model	2.2*	7	0.31	0.82	0.57
	Intercept	1349.04	1	1349.04	3528.85	< 0.001
* R^2 = 0.074, Adj. R2 = -0.016	spray treatment	0.26	3	0.09	0.23	0.88
	thrips	1.72	1	1.72	4.5	0.04
	spray treatment x thrips	0.22	3	0.073	0.19	0.9
	Error	27.53	72	0.38		
	Total	1378.76	80			
Total necrosis damage due to	Corrected Model	133521.89*	7	19074.55	45.76	< 0.001
Spray treatments	Intercept	85477.81	1	85477.81	205.04	< 0.001
	spray treatment	133295.04	3	44431.68	106.58	< 0.001
* R^2 = 0.816, Adj. R2 = 0.799	thrips	0.61	1	0.61	0.001	0.97
	spray treatment x thrips	226.24	3	75.41	0.18	0.91
	Error	30015.3	72	416.88		
	Total	249015	80			
