# Supplementary material for: Plant-oil-based adhesive formulations induce plant stress responses and enhance resistance of Chrysanthemum to western flower thrips
Source: RSC Adv. 2026 Oct 27. Online ahead of print. doi: 10.1039/d6ra07230e (PMC13711384; doi:10.1039/d6ra07230e)
Supplement: Supplementary file 1 [file RA-OLF-D6RA07230E-s001.pdf]

This journal is © The Royal Society of Chemistry

# SUPPLEMENTAL INFORMATION

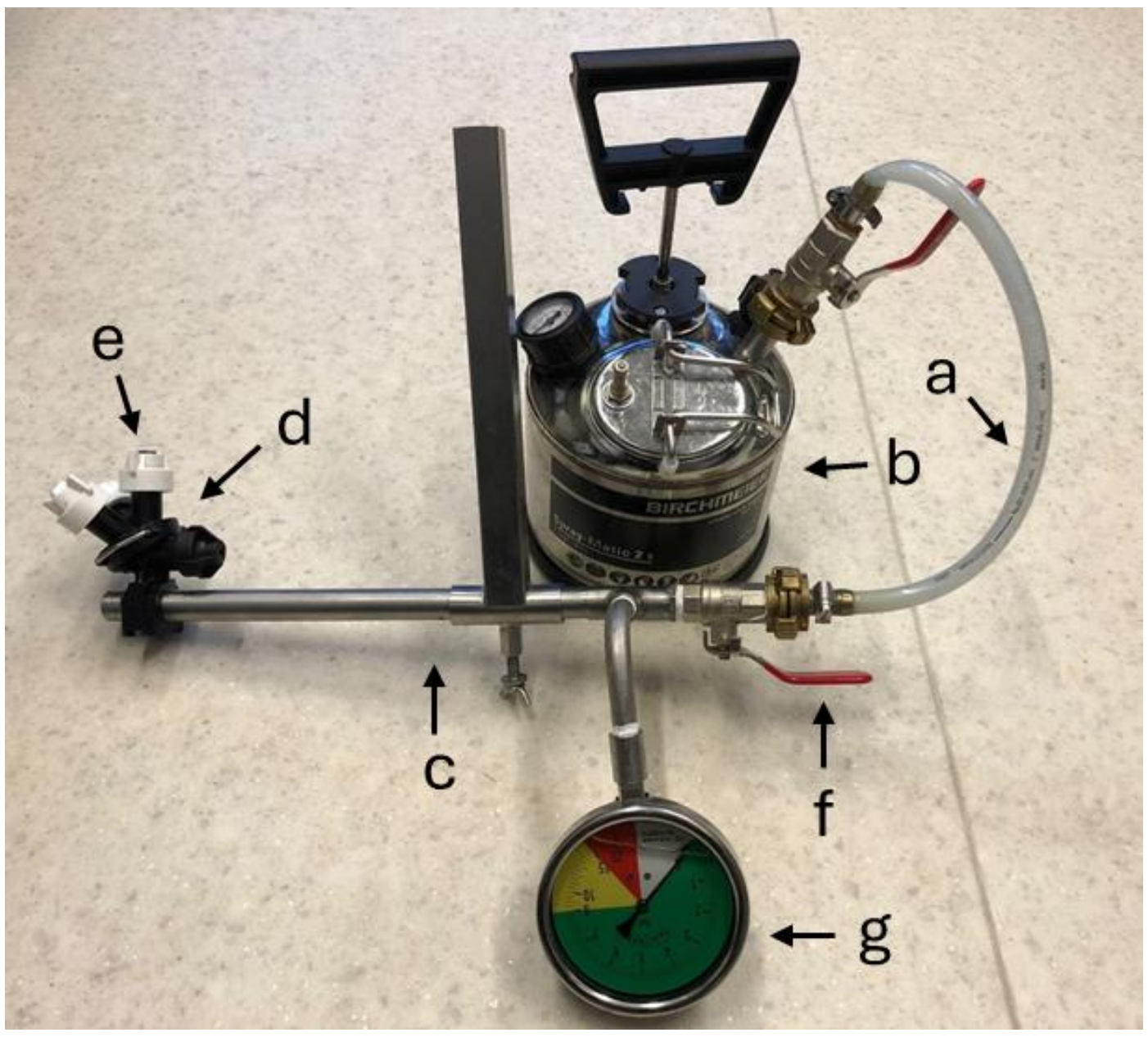


**Fig. S1** Modified Birchmeyer Spray Matic 2S (DCM Nederland B.V., Nieuw-Vennep, The Netherlands). A silicone tube (a) is used to connect the tank (b) to a metal tube (c) with connector pieces on the far end (d) to attach the Teejet 2502-SS nozzle (e) (Teejet Technologies Northwest Europe, Schorndorf, Germany). The red valves (f) on the metal tube are used to start and stop spraying while a pressure gauge (g) is used to monitor the amount of spraying pressure in bar

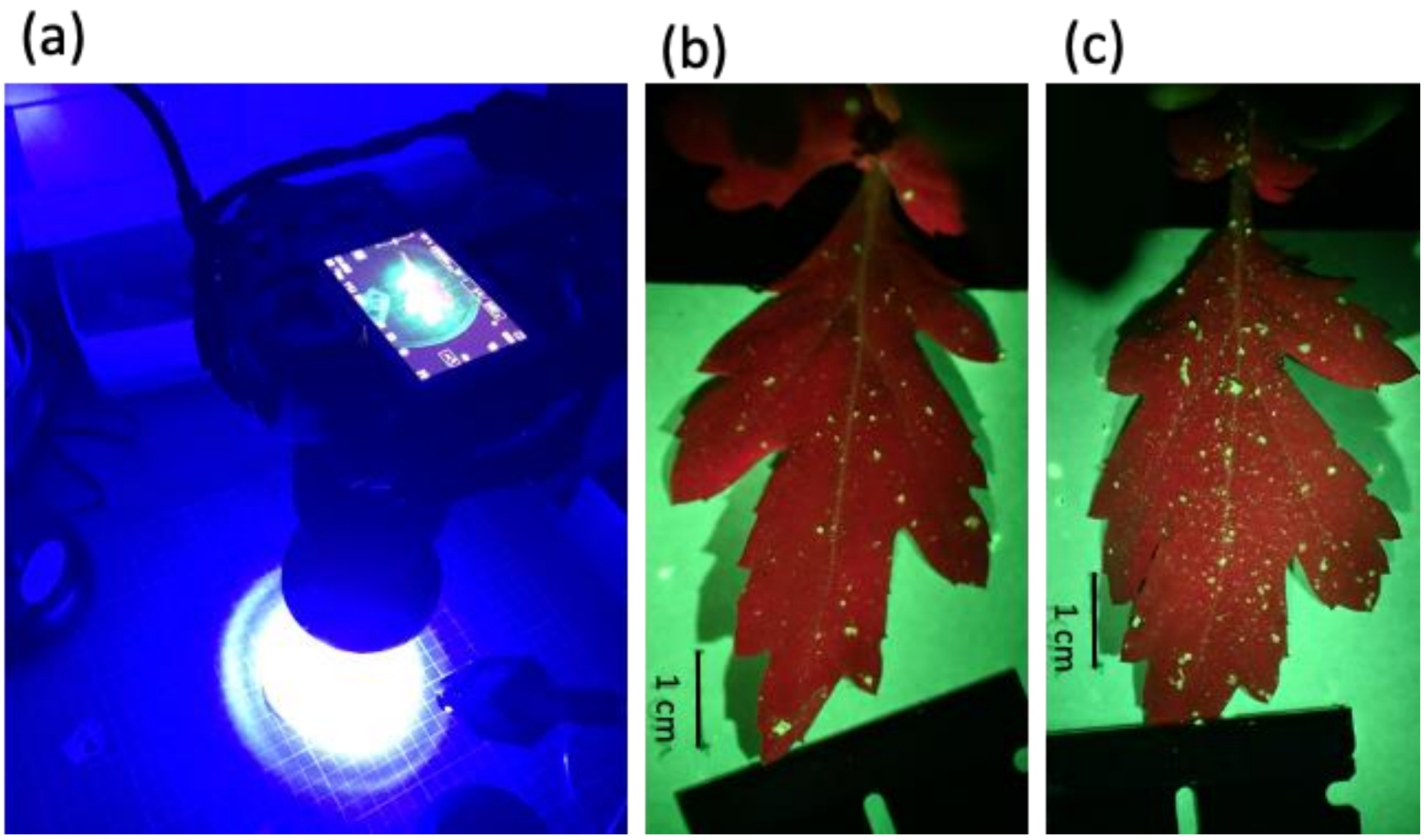


**Fig. S2** Camera setup with blue UV ligt (Nightsea[tm] Royal blue) (a), Example of coverage on the 4th leaf from the bottom of a 20 ml MIX sprayed plant (b) and on the 4th leaf of a 40 ml MIX sprayed plant (c)

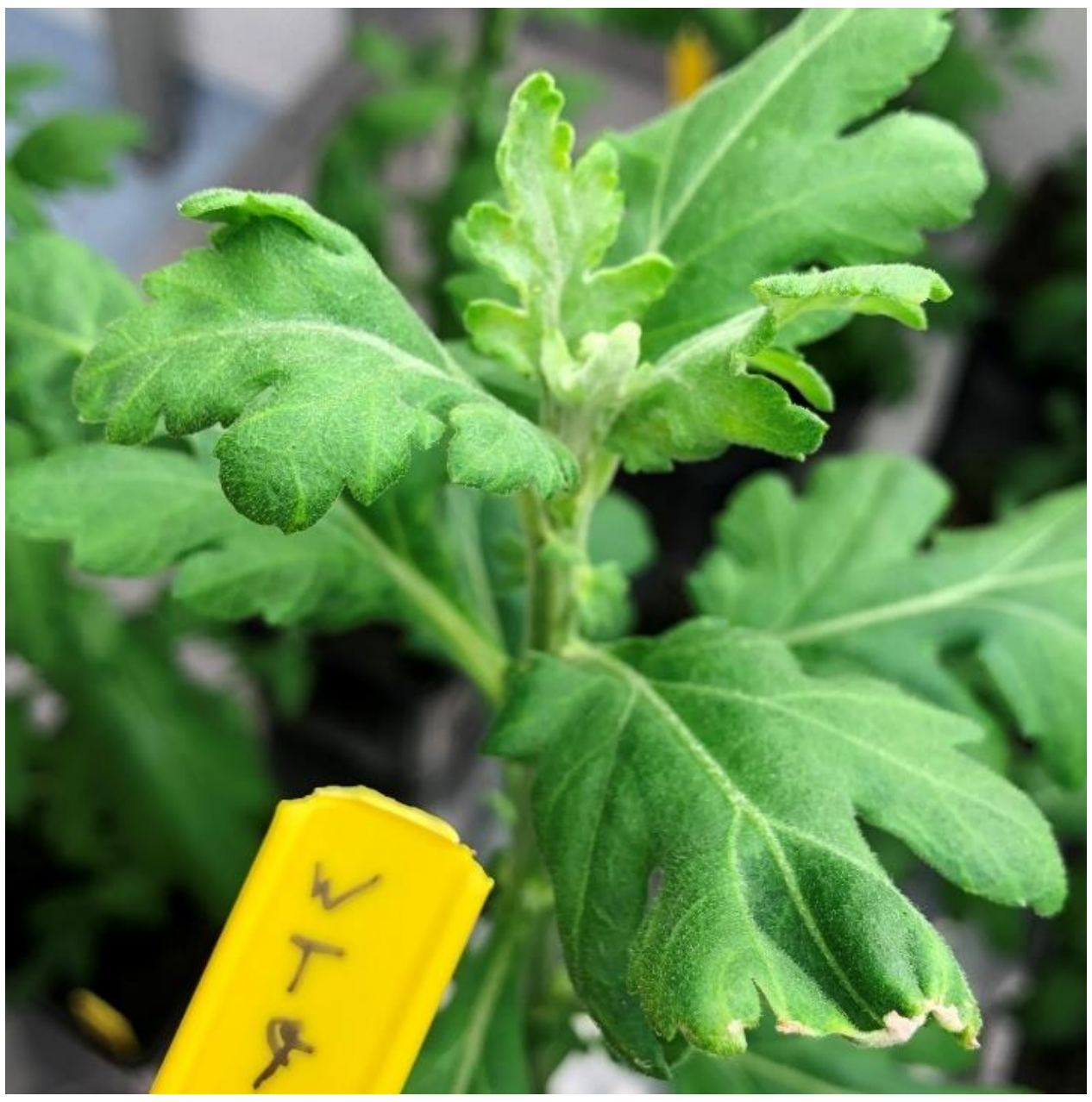

**Fig. S3** Malformed growth in the water control treatment of plant assay MIX. The development damage is caused by thrips larvae feeding in the top of the plant and / or by systemic responses of the plant to this feeding

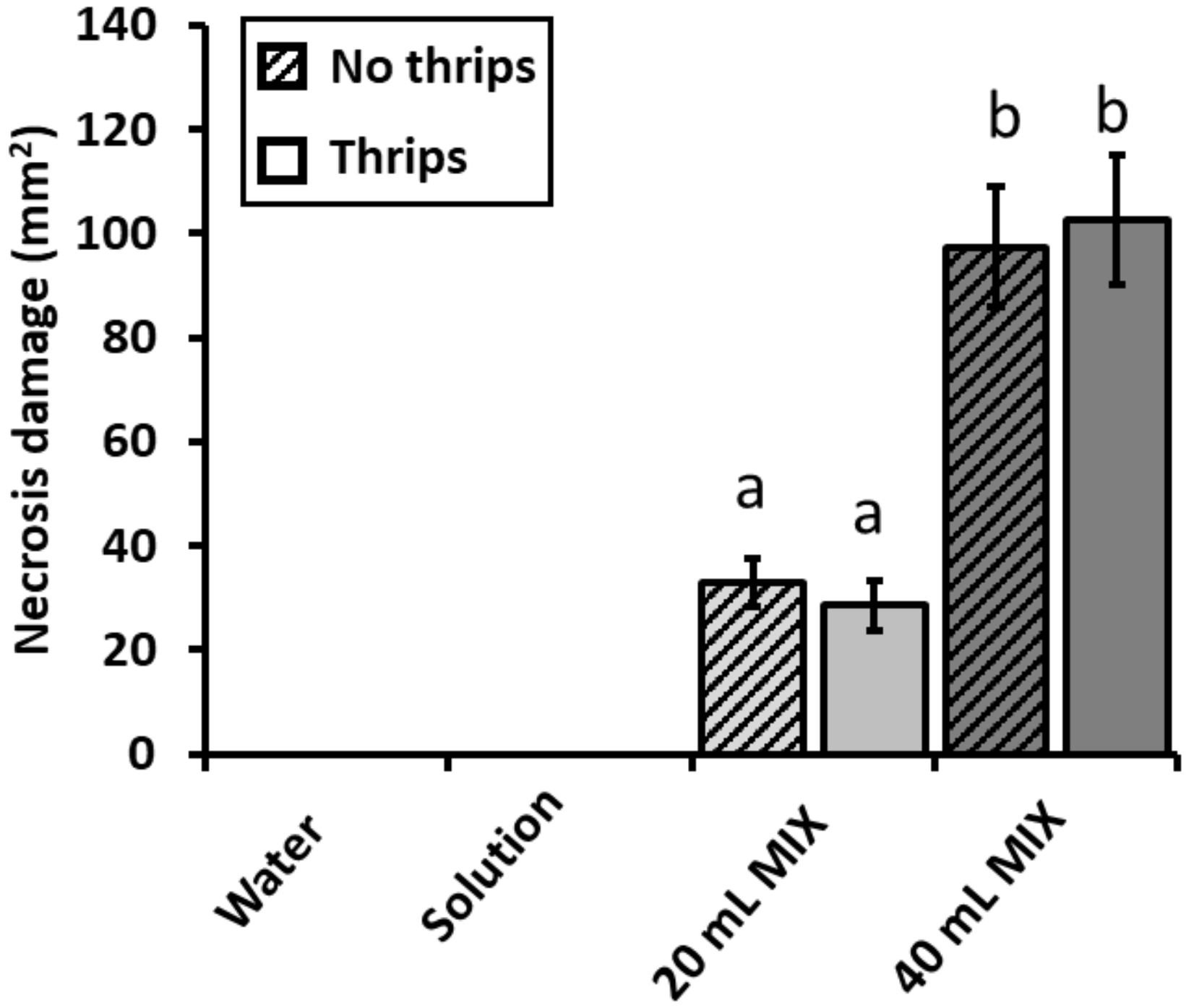


**Fig. S4** Plant assay with MIX, mean (± SE) phytotoxic (necrosis) damage in mm$^2$ of plants that were sprayed with several treatments: water, solution (water with alginate, F-108 and $Cacl_2$), 20 mL solution with adhesive MIX oil droplets and 40 mL solution with MIX droplets and infested with thrips. Letters indicate significant differences as shown by factor analysis via two-way ANOVA, ($P < 0.05$)

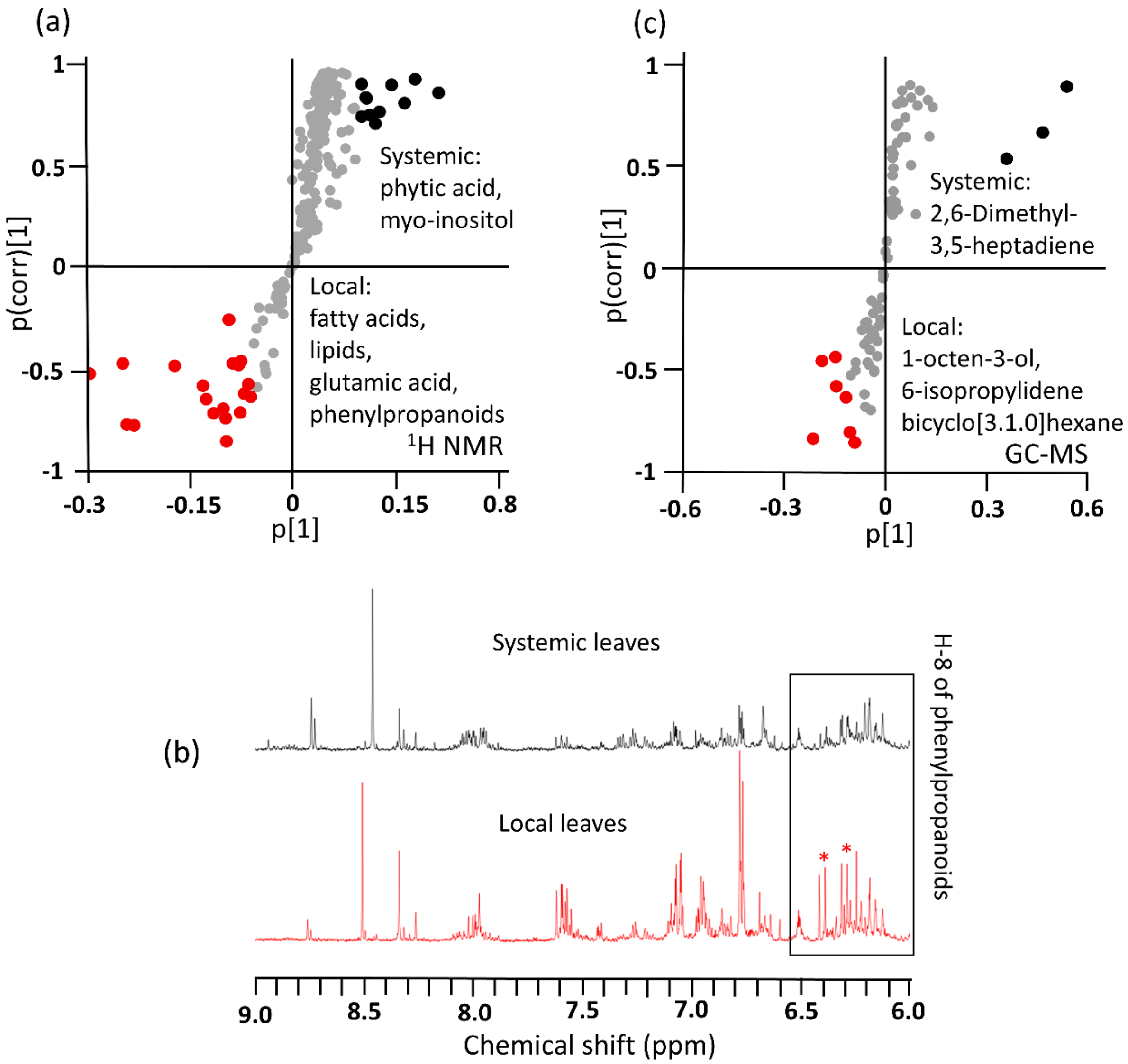


**Fig. S5** S-plot of orthogonal partial least square model including all $^{1}$H NMR data, classified in local or systemic (a), comparison of $^{1}$H NMR spectra in a characteristic phenolic region (δ6.0 – δ9.0) (b), and S-plot of orthogonal partial least square modeling of HS-GC-MS data, classified in local or systemic (c). Asterisk *: H-8 of 3,5-dicaffeoylphenylpropanoids

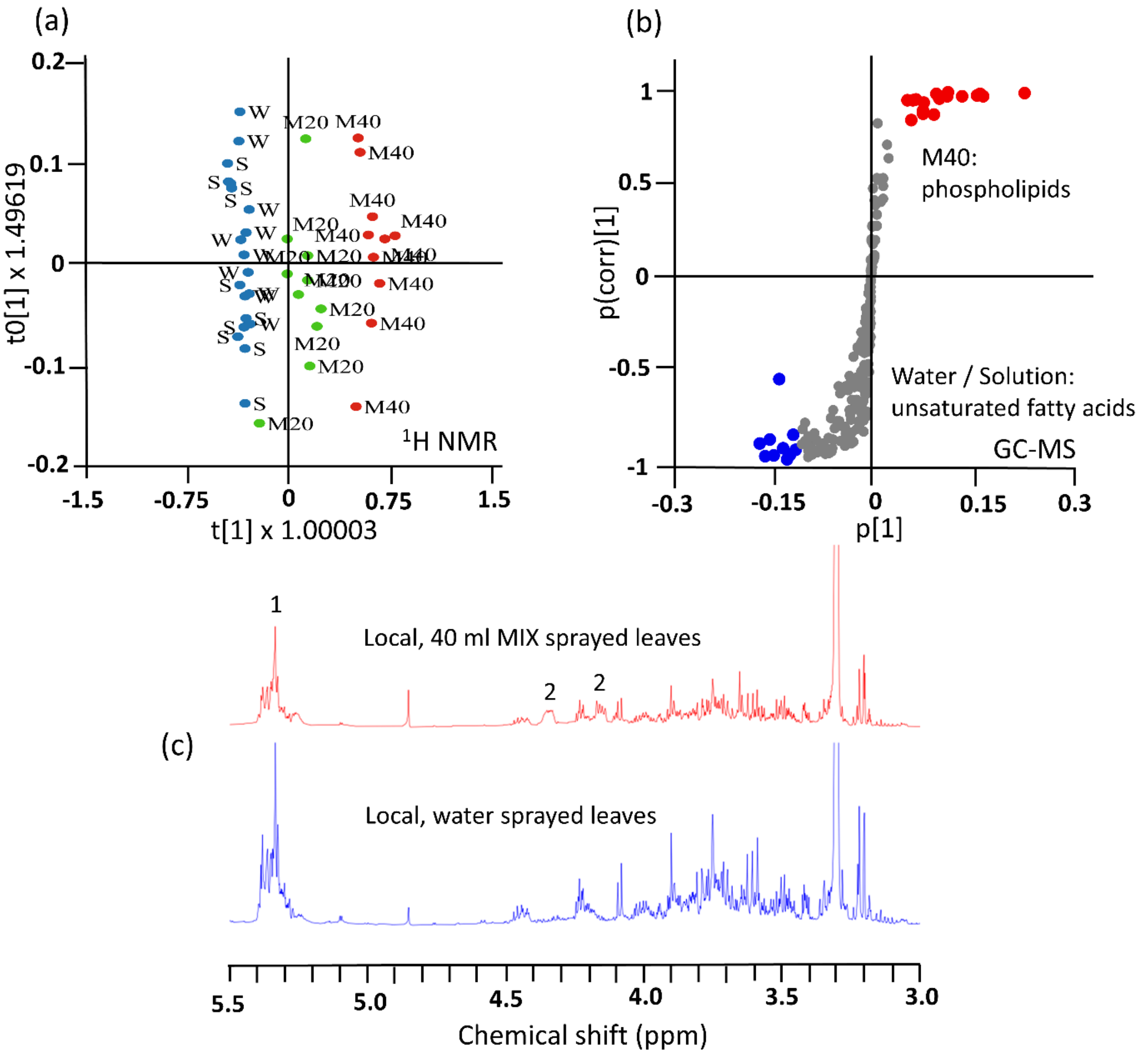


**Fig. S6** Score plot (a) and S-plot (b) of orthogonal partial least square (discriminant analysis) models of $^{1}H$ NMR dataset of local leaves, classified by spray treatment (blue: Water, "W", Solution "S", green: 20 ml MIX, "M20", red: 40 ml MIX, "M40") (n = 5), and the comparison of $^{1}H$ NMR spectra of water / Solution- and 40 ml MIX-sprayed leaves (c) in the range of δ3.0 – δ5.5 "1": $^{1}H$ resonance of unsaturated fatty acids, "2": $^{1}H$ resonances of phospholipids

**SI Table 1** Plant assay with MIX, statistics of ANOVAs

| ***Measured variable and R2*** | ***Source of Variation*** | ***SS*** | ***df*** | ***MS*** | ***F*** | ***P*** |
|---|---|---|---|---|---|---|
| Total thrips damage on sprayed | Corrected Model | 4047.88* | 3 | 1349.29 | 3.06 | 0.04 |
| leaves | Intercept | 81812.03 | 1 | 81812.03 | 185.64 | < 0.01 |
| | spray treatment | 4047.88 | 3 | 1349.29 | 3.06 | 0.04 |
| * $R^2$ = 0.203, Adj. R2 = 0.137 | Error | 15865.1 | 36 | 440.7 | | |
| | Total | 101725 | 40 | | | |
| Total thrips damage on newly | Corrected Model | 0.11* | 3 | 0.04 | 5.56 | < 0.01 |
| grown leaves | Intercept | 16.1 | 1 | 16.1 | 2545.5 | < 0.001 |
| | spray treatment | 0.29 | 3 | 0.04 | 5.56 | < 0.01 |
| * $R^2$ = 0.317, Adj. R2 = 0.260 | Error | 16.43 | 36 | 0.006 | | |
| | Total | 0.33 | 40 | | | |
| Number of thrips larvae per plant | Corrected Model | 162.6* | 3 | 54.2 | 4.34 | 0.01 |
| | Intercept | 4410 | 1 | 4410 | 353.27 | < 0.001 |
| * $R^2$ = 0.266, Adj. R2 = 0.204 | spray treatment | 162.6 | 3 | 54.2 | 4.34 | 0.01 |
| | Error | 449.4 | 36 | 12.48 | | |
| | Total | 5022 | 40 | | | |
| Number of leaves with growth | Corrected Model | 1.48* | 3 | 0.49 | 0.44 | 0.73 |
| damage due to thrips feeding | Intercept | 1677.03 | 1 | 1677.03 | 1490.7 | < 0.001 |
| per plant | spray treatment | 1.48 | 3 | 0.49 | 0.44 | 0.73 |
| | Error | 40.5 | 36 | 1.13 | | |
| * $R^2$ = 0.035, Adj. R2 = -0.045 | Total | 1719 | 40 | | | |

**SI Table 2** Plant assay with MIX, statistics of Two-way ANOVAs

| ***Measured variable and R2*** | ***Source of Variation*** | ***SS*** | ***df*** | ***MS*** | ***F*** | ***p*** |
|---|---|---|---|---|---|---|
| *Chrysanthemum* shoot length | Corrected Model | 131.05* | 7 | 18.72 | 10.06 | < 0.001 |
| | Intercept | 155849.51 | 1 | 155849.5 | 83753.8 | < 0.001 |
| * $R^2$ = 0.494, Adj. R2 = 0.445 | spray treatment | 8.39 | 3 | 2.8 | 1.5 | 0.22 |
| | thrips | 117.61 | 1 | 117.61 | 63.21 | < 0.001 |
| | spray treatment x thrips | 5.04 | 3 | 1.68 | 0.9 | 0.44 |
| | Error | 133.98 | 72 | 1.86 | | |
| | Total | 156114.54 | 80 | | | |
| *Chrysanthemum* shoot dry weight | Corrected Model | 2.2* | 7 | 0.31 | 0.82 | 0.57 |
| | Intercept | 1349.04 | 1 | 1349.04 | 3528.85 | < 0.001 |
| * $R^2$ = 0.074, Adj. R2 = -0.016 | spray treatment | 0.26 | 3 | 0.09 | 0.23 | 0.88 |
| | thrips | 1.72 | 1 | 1.72 | 4.5 | 0.04 |
| | spray treatment x thrips | 0.22 | 3 | 0.073 | 0.19 | 0.9 |
| | Error | 27.53 | 72 | 0.38 | | |
| | Total | 1378.76 | 80 | | | |
| Total necrosis damage due to | Corrected Model | 133521.89* | 7 | 19074.55 | 45.76 | < 0.001 |
| Spray treatments | Intercept | 85477.81 | 1 | 85477.81 | 205.04 | < 0.001 |
| | spray treatment | 133295.04 | 3 | 44431.68 | 106.58 | < 0.001 |
| * $R^2$ = 0.816, Adj. R2 = 0.799 | thrips | 0.61 | 1 | 0.61 | 0.001 | 0.97 |
| | spray treatment x thrips | 226.24 | 3 | 75.41 | 0.18 | 0.91 |
| | Error | 30015.3 | 72 | 416.88 | | |
| | Total | 249015 | 80 | | | |
